# Zinc transporter ZIP13 suppresses beige adipocyte biogenesis and energy expenditure by regulating C/EBP-β expression

**DOI:** 10.1371/journal.pgen.1006950

**Published:** 2017-08-30

**Authors:** Ayako Fukunaka, Toshiyuki Fukada, Jinhyuk Bhin, Luka Suzuki, Takamasa Tsuzuki, Yuri Takamine, Bum-Ho Bin, Toshinori Yoshihara, Noriko Ichinoseki-Sekine, Hisashi Naito, Takeshi Miyatsuka, Shinzaburo Takamiya, Tsutomu Sasaki, Takeshi Inagaki, Tadahiro Kitamura, Shingo Kajimura, Hirotaka Watada, Yoshio Fujitani

**Affiliations:** 1 Department of Metabolism & Endocrinology, Juntendo University Graduate School of Medicine, Tokyo, Japan; 2 Laboratory of Developmental Biology & Metabolism, Institute for Molecular & Cellular Regulation, Gunma University, Maebashi, Gunma, Japan; 3 AMED-JST-CREST Program, Tokyo, Japan; 4 Faculty of Pharmaceutical Sciences, Tokushima Bunri University, Tokushima, Japan; 5 Division of Pathology, Department of Oral Diagnostic Sciences, School of Dentistry, Showa University, Tokyo, Japan; 6 RIKEN Center for Integrative Medical Sciences, Yokohama, Japan; 7 Department of Molecular Carcinogenesis, The Netherlands Cancer Institute, Amsterdam, The Netherlands; 8 Graduate School of Health and Sports Science, Juntendo University, Inzai, Chiba, Japan; 9 Department of Tropical Medicine and Parasitology, Graduate School of Medicine, Juntendo University, Tokyo, Japan; 10 Laboratory of Metabolic Signaling, Institute for Molecular & Cellular Regulation, Gunma University, Maebashi, Gunma, Japan; 11 Laboratory of Epigenetics and Metabolism, Institute for Molecular & Cellular Regulation, Gunma University, Maebashi, Gunma, Japan; 12 UCSF Diabetes Center and Department of Cell and Tissue Biology, University of California–San Francisco, San Francisco, United States of America; 13 PRESTO-JST, Tokyo, Japan; 14 Center for Identification of Diabetic Therapeutic Targets, Juntendo University Graduate School of Medicine, Tokyo, Japan; 15 Sportology Center, Juntendo University Graduate School of Medicine, Tokyo, Japan; 16 Center for Therapeutic Innovations in Diabetes, Juntendo University Graduate School of Medicine, Tokyo, Japan; Stanford University School of Medicine, UNITED STATES

## Abstract

Given the relevance of beige adipocytes in adult humans, a better understanding of the molecular circuits involved in beige adipocyte biogenesis has provided new insight into human brown adipocyte biology. Genetic mutations in *SLC39A13/ZIP13*, a member of zinc transporter family, are known to reduce adipose tissue mass in humans; however, the underlying mechanisms remains unknown. Here, we demonstrate that the *Zip13*-deficient mouse shows enhanced beige adipocyte biogenesis and energy expenditure, and shows ameliorated diet-induced obesity and insulin resistance. Both gain- and loss-of-function studies showed that an accumulation of the CCAAT/enhancer binding protein-β (C/EBP-β) protein, which cooperates with dominant transcriptional co-regulator PR domain containing 16 (PRDM16) to determine brown/beige adipocyte lineage, is essential for the enhanced adipocyte browning caused by the loss of ZIP13. Furthermore, ZIP13-mediated zinc transport is a prerequisite for degrading the C/EBP-β protein to inhibit adipocyte browning. Thus, our data reveal an unexpected association between zinc homeostasis and beige adipocyte biogenesis, which may contribute significantly to the development of new therapies for obesity and metabolic syndrome.

## Author summary

Inducible brown fat-like cells, named beige adipocytes have recently been a topic of great interest, mainly because they are induced in response to external cues, and are closely associated with adult human brown adipocyte. Therefore, the identification of selective molecular circuits involved in beige adipocyte biogenesis and thermogenesis will enable the selective induction of white adipocyte browning as a therapy for obesity. Here, we show that zinc homeostasis, which is controlled by ZIP13, a protein associated with human disease, is essential for the accurate regulation of beige adipocyte differentiation. Inhibition of ZIP13 function enhances beige adipocyte biogenesis and thermogenesis, highlighting the potential of ZIP13 as a therapeutic target for obesity and metabolic syndrome.

## Introduction

Obesity and its associated metabolic diseases are caused by a long-term imbalance between energy intake and energy expenditure. Adipose tissue, a major factor in controlling the balance of energy, is composed of white and brown adipocytes, which have two distinct functions: white adipocytes store excess energy, whereas brown adipocytes specialize in expending energy. The unique metabolic properties of brown adipocytes depend on their mitochondrial density, fuel oxidation capacity, and exclusive expression of uncoupling protein-1 (UCP1). Inducible brown fat-like cells, named beige adipocytes, have also been identified in white adipose tissue (WAT). Beige adipocytes are induced by various external cues, such as chronic cold exposure, long-term treatment with a peroxisome proliferator-activated receptor (PPAR)-γ agonist, cancer cachexia, and bariatric surgery [1–3]. The presence and activity of thermogenic adipocytes (brown and beige adipocytes) are associated with improved global metabolic fitness, such as improvements in insulin resistance and glucose homeostasis [2,4,5]; conversely, thermogenic adipocytes decrease with age and obesity in mice and humans [6,7]. Recent studies indicate that adult human brown adipose tissue (BAT) in the supraclavicular region has beige-like characteristics [8–11]. These findings indicate the potential importance of beige fats in human obesity and metabolic disease. Therefore, identifying a selective molecular pathway that regulates the acquisition of beige adipocyte properties will enable us to selectively and preferentially promote beige adipocyte biogenesis and thermogenesis as a therapy for obese or older subjects who do not have active BAT depots [1].

Identifying and implementing therapies based on beige fat requires a detailed understanding of the differences in the developmental mechanisms and functions of white, brown, and beige adipocytes. Differentiation mechanisms of all of these fat cell types share many transcriptional regulators, such as PPARγ and members of the CCAAT/enhancer binding protein (C/EBP) family of transcription factors [12]. C/EBP-β is induced in the early phase of adipogenesis and is crucial for activating PPARγ expression. PPARγ collaborates with C/EBP-α to bind and regulate the expression of most adipocyte-associated genes, including *aP2*, in fat cells [13]. Many transcription factors that direct cells toward a brown/beige adipocyte identity rather than a white adipocyte identity act by modulating the core adipogenic transcriptional machinery. PRDM16, which is one of the most important transcriptional co-regulators of brown/beige adipocyte differentiation [14], promotes a brown fat-selective gene program, mainly via protein-protein interactions with transcriptional factors such as C/EBP-β, PPARγ, and zinc finger protein 516 (Zfp516) [3,15,16].

Zinc is an essential nutrient for all living organisms and is required for the structure and function of a wide range of proteins; 10% of all human proteins have the potential to bind zinc [17]. Zinc acts as both an intracellular and extracellular signaling effector; zinc signaling is mediated by zinc transporters and metallothioneins that regulate various cellular functions [18]. Cellular zinc homeostasis is tightly regulated by two families of zinc transporter proteins, namely, the zinc transporter (ZnT) family, which controls zinc efflux out of the cytosol, and the Zrt/Irt-related protein (ZIP) family, which controls zinc influx into the cytosol [19]. Dysfunction of zinc signaling leads to physiological disturbances. For example, our group and others showed that extracellular zinc signaling via the zinc transporter ZnT8 regulates hepatic insulin clearance, and that altered ZnT8 function is involved in type 2 diabetes pathogenesis [20,21], indicating that the precise control of zinc homeostasis is crucial for maintaining health and preventing diseases, including lifestyle-associated diseases. Intriguingly, zinc deficiency significantly reduces the DNA-binding activity of PPARs [22], suggesting that some zinc-containing proteins that participate in brown/beige adipocyte differentiation and function (e.g., PRDM16, Kruppel-like factor 11 (KLF11), and Zfp516) might be dysregulated by changes in zinc homeostasis.

In this study, we focused on the zinc transporter ZIP13 because *Zip13*-deficient (*Zip13*-KO) mice and humans with spondylocheirodysplastic Ehlers-Danlos syndrome who carry a loss-of-function mutation in *SLC39A13* have been reported to have a significantly decreased white fat mass [23]. We show that ZIP13 is a crucial regulator of beige adipocyte differentiation, and negatively regulates C/EBP-β protein levels, illustrating the physiological relevance of the ZIP13-C/EBP-β axis in beige adipocyte biogenesis and thermogenesis, and its therapeutic potential in obesity treatment.

## Results

### Inguinal fat tissue browning is accelerated in *Zip13-*KO mice

**Fig 1:**
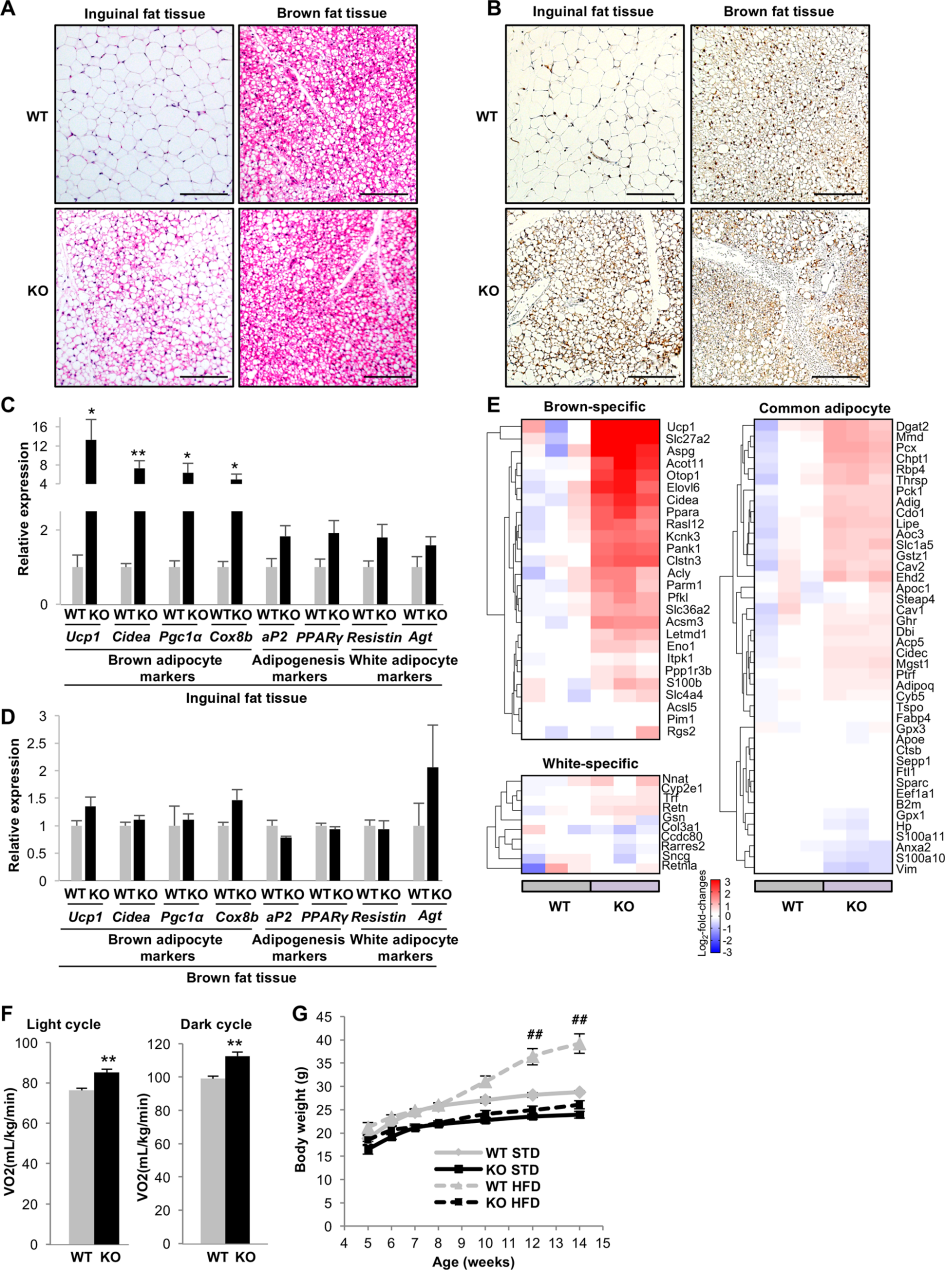
Upregulation of inguinal fat tissue browning and O_2_ consumption rate in *Zip13*-KO mice. (A) H & E staining of inguinal fat and brown fat tissue in 10-week-old WT and *Zip13*-KO mice. Scale bars = 100 μm. (B) Immunohistochemical staining of the UCP1 in inguinal fat and brown fat tissue sections from 10-week-old WT and *Zip13*-KO mice. Scale bars = 100 μm. (C) Expression of the indicated genes in the inguinal fat tissue of 10-week-old WT and *Zip13*-KO mice (n = 5–6). (D) Expression of the indicated genes in the brown fat tissue of 10-week-old WT and *Zip13*-KO mice (n = 5–6). (E) Heat map of mRNA levels of brown fat-specific, white fat-specific, and common fat genes in the iWAT from 10-week-old WT and *Zip13*-KO mice (n = 3). (F) Energy expenditure of 10-week-old WT and *Zip13*-KO mice during the light (left) or dark cycle (right) (n = 4–6). (G) Body weights of mice from 5 to 14 weeks of age when fed a standard (STD) or high-fat diet (HFD) (n = 7–9). Error bars show SEM. *p < 0.05, **p < 0.01 (WT vs. *Zip13*-KO), ^##^*p* < 0.01 (WT STD vs. WT HFD).

We previously reported that ZIP13 may be involved in adipose tissue homeostasis [23]. To clarify this point, we first weighed the WAT and BAT of *Zip13*-KO mice and their wild-type (WT) littermates (control). As shown in S1A Fig, the interscapular BAT and inguinal WAT (iWAT) weights were similar in *Zip13*-KO and WT mice, but the epididymal WAT (eWAT) weight was significantly lower in *Zip13*-KO mice. *Zip13* expression was higher in the eWAT and BAT than in the iWAT (S1B Fig). Surprisingly, hematoxylin and eosin (H & E) staining of the iWAT in *Zip13*-KO mice showed large clusters of cells with multilocular lipid droplets, which are characteristic of the browning of iWAT depots (Fig 1A). Consistently, immunohistochemical staining revealed a high number of UCP1-positive cells in the *Zip13*-KO iWAT (Fig 1B); gene expression analyses confirmed that brown fat genes were significantly upregulated in the *Zip13*-KO iWAT (Fig 1C). There were no significant differences between WT and *Zip13*-KO mice in eWAT and BAT morphology, or in the expression of various brown adipocyte markers in BAT (Fig 1A and 1D, S1C Fig); however, we observed that the expression of brown adipocyte markers in the eWAT of *Zip13*-KO mice tended to be higher than that of WT mice, although statistical significance was not observed (S1D Fig).

We assessed overall gene expression changes in the iWAT by microarray analyses of RNAs isolated from the iWATs of *Zip13*-KO mice and their WT littermates, and identified differentially expressed genes (1,260 upregulated and 1,082 downregulated genes in *Zip13*-KO mice compared with WT mice) (S2A Fig and S1 Table). Gene Ontology Biological Process (GOBP) analysis and Kyoto Encyclopedia of Genes and Genomes (KEGG) pathway enrichment analysis revealed that genes involved in inflammatory responses were downregulated, whereas genes involved in fatty acid metabolism and mitochondrial function were upregulated in the *Zip13*-KO iWAT, suggesting that an accelerated adipocyte-browning process occurs in the iWAT of *Zip13*-KO mice (S2B and S2C Fig, S2 Table). Pan et al. identified brown fat-specific, white fat-specific, and common fat genes by the RNA-sequencing of BAT, eWAT, and soleus-muscle tissue [24]. We used these criteria to profile the gene expression patterns in the *Zip13*-KO iWAT, and demonstrated the upregulation of a broad spectrum of brown fat-specific genes, a slight upregulation in common fat genes, and no change in white fat-specific genes (Fig 1E). Furthermore, the oxygen consumption rate (OCR) was significantly higher in the inguinal fat tissue of *Zip13*-KO mice than that of WT mice (S1E Fig), although there was no significant difference of OCR in brown fat tissue between the two groups. These results indicate that there was an increase in the number of functional beige adipocytes in *Zip13*-KO inguinal fat tissue.

### O_2_ consumption rate is elevated in *Zip13-*KO mice

The increase in beige adipocyte characteristics in *Zip13*-KO mice prompted us to examine the metabolic profiles of these mice. Indeed, we observed a significantly higher oxygen consumption (VO_2_) rate in *Zip13*-KO mice than in WT mice under both light and dark conditions at 23°C (Fig 1F), without any changes in food intake (S3A Fig). Furthermore, the locomotor activity of the *Zip13*-KO mice tended to decrease (S3B Fig), although this change was not statistically significant. These results suggested that the increase in VO_2_ in *Zip13*-KO mice was not due to hyperactivity or impaired food intake. To further investigate the role of ZIP13 in thermogenesis, we measured VO_2_ in thermoneutral conditions (30°C) after activation of the β3-adrenoreceptor agonist [25]. As shown in S3C Fig, VO_2_ levels of *Zip13*-KO mice were significantly increased after the administration of β3-adrenoreceptor agonist CL316,243, suggesting that ZIP13 might play a role in thermogenesis. We subsequently examined whether *Zip13*-KO mice acquired resistance to high-fat diet (HFD)-induced obesity. Body weights of WT mice were significantly increased by the HFD compared with a standard diet (STD); however, *Zip13*-KO mice were resistant to HFD-induced obesity primarily due to low fat mass gain (Fig 1G, S4A and S5A–S5E Figs). Furthermore, *Zip13*-KO mice appeared to have an improved glucose tolerance and insulin tolerance, compared to WT mice (S4B and S5D Figs). Taken together, these results indicated that *Zip13* deletion *in vivo* promotes beige adipocyte biogenesis and energy expenditure, and thereby reduces diet-induced obesity and insulin resistance.

### ZIP13 negatively regulates adipocyte browning in a cell-autonomous manner

**Fig 2:**
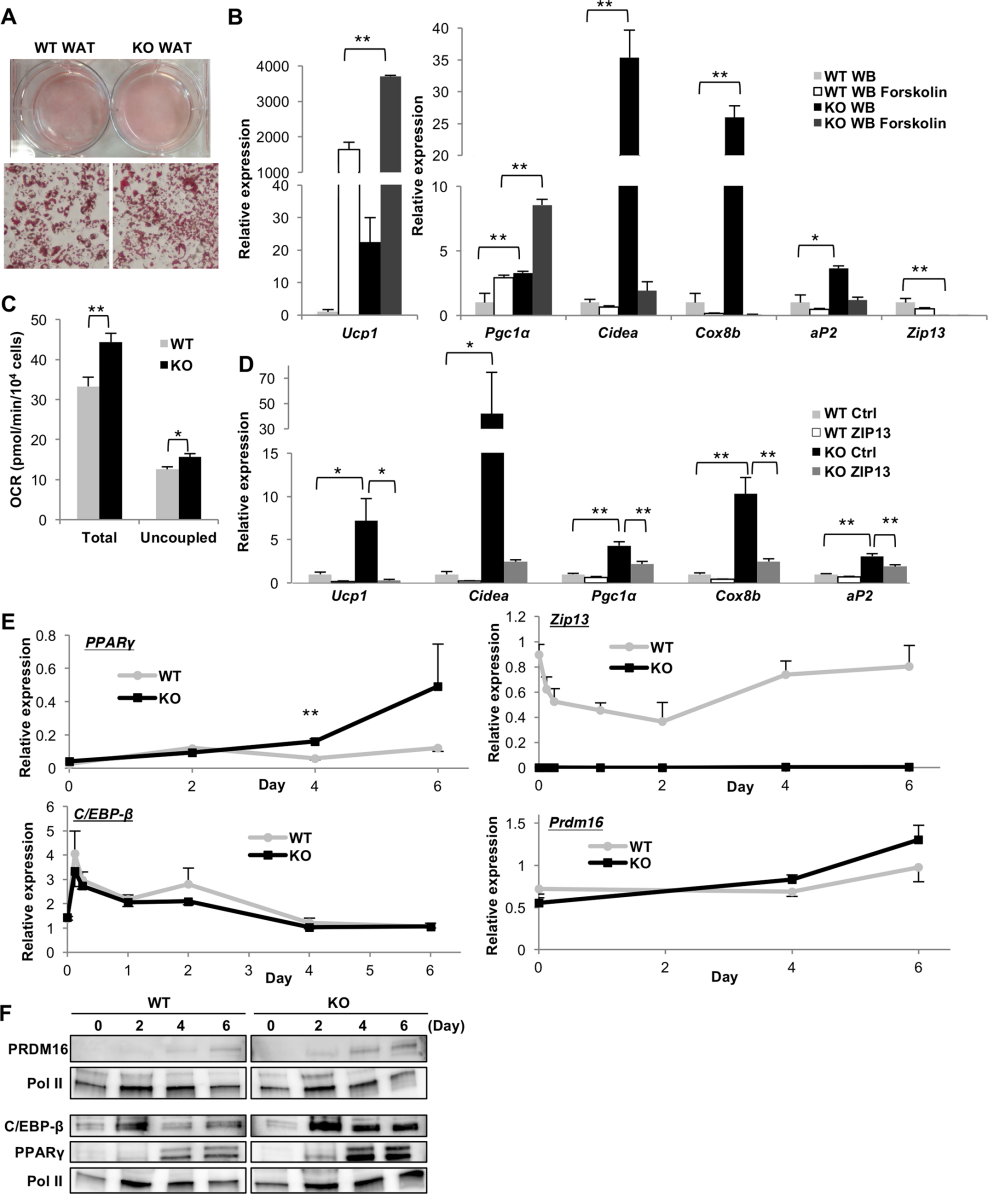
Adipocyte browning is accelerated in white preadipocytes from *Zip13*-KO mice. (A) Oil Red O staining of preadipocytes from WT and *Zip13*-KO mice in pro-adipogenic conditions. (B) Expression levels of the indicated genes in differentiated adipocytes in the presence or absence of forskolin (n = 3). (C) Total and uncoupled (oligomycin-insensitive) respiration of differentiated adipocytes (n = 3). (D) Differentiation of white preadipocytes from WT and *Zip13*-KO mice expressing an empty vector (Ctrl) or ZIP13-HA (ZIP13); mRNA levels of the indicated genes were measured using qRT-PCR (n = 4). (E) Time course of mRNA expression in differentiated white preadipocytes from WT and *Zip13*-KO mice (n = 3). (F) Time course of protein expression in WT and *Zip13*-KO preadipocytes after differentiation. Nuclear fractions were analyzed by immunoblotting. RNA Pol II was included as a loading control. Error bars show SEM. *p < 0.05, **p < 0.01.

To clarify whether the increase in iWAT browning in *Zip13*-KO mice occurs in a cell-autonomous manner, primary white preadipocytes isolated from the iWAT of WT or *Zip13*-KO mice were cultured to induce their differentiation into adipocytes under defined conditions [26–28]. We found higher gene expression levels of several brown adipocyte markers and an adipogenesis marker (*aP2*) (Fig 2A and 2B), and higher total and oligomycin-insensitive cellular respiration in *Zip13*-KO cells than in WT cells (Fig 2C), suggesting that preadipocytes from *Zip13*-KO mice cell-autonomously accelerate adipocyte browning. Furthermore, treatment with the cAMP-inducer forskolin also increased the expression of thermogenic genes, including *Ucp1* and *Pgc1*α, in *Zip13*-KO cells (Fig 2B), indicating that the differentiated cells were functional. Importantly, the exogenous expression of ZIP13 in *Zip13*-KO cells efficiently repressed the expression of brown adipocyte markers and the adipogenesis marker (Fig 2D, S6A Fig). Taken together, these results clearly demonstrate that ZIP13 negatively and cell-autonomously regulates adipocyte browning.

### C/EBP-β protein levels are increased in *Zip13-*KO cells

To investigate the mechanism by which ZIP13 regulates adipocyte browning, we immortalized white preadipocytes from *Zip13*-KO and WT mice for further experiments. Stimulation with a cocktail that induces browning of adipocytes increased the expression of the adipogenic transcription factor *PPARγ* in *Zip13*-KO cells (Fig 2E). Since PPARγ is positively regulated by the transcription factors C/EBP-β and C/EBP-δ [29], we next examined C/EBP-β and C/EBP-δ gene and protein expression levels in these cells. Although the mRNA levels of *C/EBP-β* and *C/EBP-δ* genes were expressed comparably in *Zip13*-KO and WT cells (Fig 2E, S6B Fig), the C/EBP-β protein level in *Zip13*-KO cells was higher than that in WT cells at 2 days after inducing differentiation, before the upregulation of PPARγ protein (Fig 2F). The expression of other early transcriptional regulators, such as *Krox20*, did not differ between WT and *Zip13*-KO cells (S6B Fig). C/EBP-β is not only important in adipogenesis, but is also essential for brown fat development; C/EBP-β cooperates with the coregulatory protein PRDM16 to act as a crucial molecular switch in determination of brown fat cell fate [15]. Intriguingly, PRDM16 protein levels also increased in *Zip13*-KO cells after 4 days of differentiation (Fig 2F), suggesting that ZIP13 is involved in the homeostatic regulation of C/EBP-β and the PRDM16 proteins, both of which are essential for adipocyte browning at either the post-transcriptional or translational level.

### *Zip13-*KO cells resemble preadipocytes expressing high levels C/EBP-β protein

**Fig 3:**
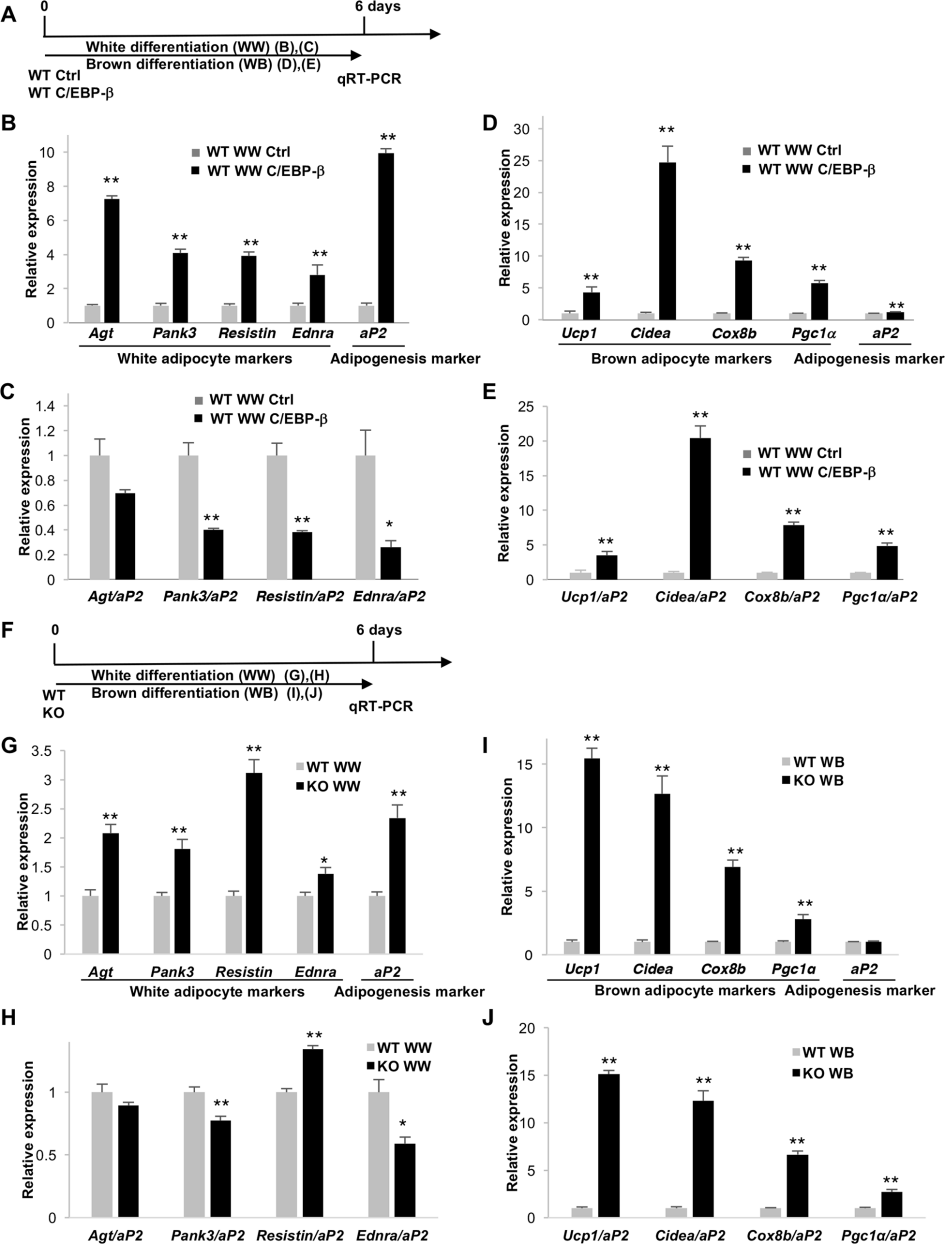
C/EBP-β overexpression accelerates adipocyte browning independently of adipogenesis. (A) Diagram showing the time course used in the following experiments (B-E) using WT white preadipocytes expressing a control vector (WT Ctrl) or HA-C/EBP-β (WT C/EBP-β). These cells were differentiated using a white adipogenic cocktail (WW) or a brown adipogenic cocktail (WB). (B) Expression of the indicated genes was measured by qRT-PCR (n = 4). (C) The mRNA levels of white adipocyte markers related to (B) were normalized to that of *aP2* (n = 4). (D) Expression levels of the indicated genes were measured by qRT-PCR (n = 4). (E) The mRNA levels of brown adipocyte markers related to (D) were normalized to that of *aP2* (n = 4). (F) Schematic of the time course used in (G-J) using WT (WT) and *Zip13*-KO (KO) white preadipocytes. (G) Expression of the indicated genes was measured using qRT-PCR (n = 4). (H) The mRNA levels for white adipocyte markers related to (G) were normalized to that of *aP2* (n = 4). (I) Expression of the indicated genes was measured using qRT-PCR (n = 4). (J) The mRNA levels of brown adipocyte markers related to (I) were normalized to that of *aP2* (n = 4). Error bars show SEM. *p < 0.05, **p < 0.01.

Since C/EBP-β is required in both white and brown adipocyte differentiation [12], we next investigated whether the regulatory expression of C/EBP-β in WT white preadipocytes might affect white versus beige adipocyte differentiation. When white preadipocytes stably expressing C/EBP-β (WPreCβ cells) were differentiated using a cocktail that induces white adipocytes, several white adipocyte markers and the adipogenesis marker *aP2* were significantly increased (Fig 3A and 3B, S7, S8A and S8B Figs). However, this effect was due to the enhancement of adipogenesis *per se*, since white adipocyte marker genes were expressed at almost the same (or at a decreased) level between control and WPreCβ cells when the mRNA levels of these genes were normalized to that of *aP2* (Fig 3C, S8C Fig). In contrast, when these cells were exposed to a brown adipogenic differentiation cocktail, brown adipocyte markers were significantly increased in WPreCβ cells (Fig 3D, S8B Fig), and this was not likely to be due to enhanced adipogenesis, since this upregulation of brown adipocyte markers was still evident after normalizing the mRNA levels of these genes to those of *aP2* (Fig 3E, S8C Fig). These results indicate that C/EBP-β, an intrinsic transcription factor that positively regulates adipogenesis, facilitates beige adipocyte differentiation. These observations led us to further assess and compare the characteristics of *Zip13*-KO and WPreCβ cells, since *Zip13*-KO cells accumulate the C/EBP-β protein (Fig 2F). In fact, we found that both white and brown adipocyte markers were increased in *Zip13*-KO cells (Fig 3F–3G and 3I, S8D and S8E Fig), and the enhanced expression of brown adipocyte markers in *Zip13*-KO cells was still evident when normalized to the expression of *aP2* (Fig 3H and 3J). Taken together, these results suggest that *Zip13*-KO cells have similar features to those of preadipocytes, which contain high levels of the C/EBP-β protein.

### ZIP13-mediated C/EBP-β degradation pathway negatively regulates adipocyte browning

**Fig 4:**
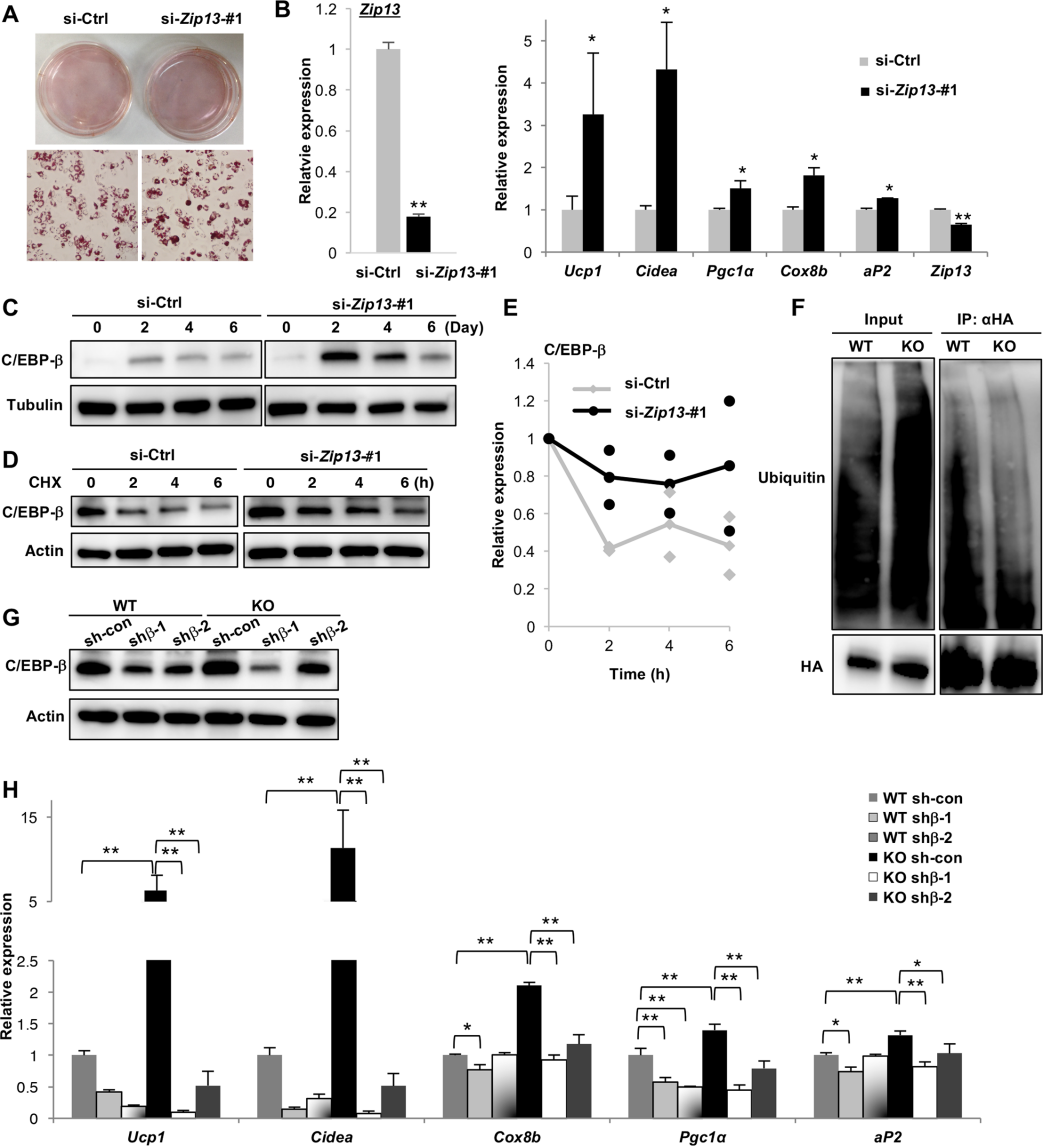
ZIP13 negatively regulates adipocyte browning by stabilizing C/EBP-β. (A) C3H10T1/2 cells transfected with an siRNA targeting *Zip13* (si-*Zip13*-#1) or a non-targeting control (si-Ctrl) were stained with Oil Red O after induction of adipocyte differentiation. (B) Left panel: *Zip13* expression after the 2.5 days of transfection; Right panel: Expression of the indicated genes was measured using qRT-PCR (n = 4). (C) Protein expression of C/EBP-β. Tubulin was used as a loading control. (D) Protein expression of C/EBP-β in the presence of CHX. C3H10T1/2 cells were transfected with the si-*Zip13*-#1 or si-Ctrl oligonucleotide; β-actin is shown as a loading control. (E) C/EBP-β protein levels were quantified by normalization to the protein level at 0 h. Each dot shows two independent experiment results and lines show the average of the experiments. (F) HA-C/EBP-β immunoprecipitation, followed by immunoblotting to detect ubiquitin. (G) Protein expression of C/EBP-β in WT and *Zip13*-KO preadipocytes expressing scramble control (sh-con) or shRNA targeting C/EBP-β (shβ-1, or shβ-2); β-actin is shown as a loading control. (H) Expression of the indicated genes, measured by qRT-PCR (n = 4). Error bars show SEM. *p < 0.05, **p < 0.01.

To further validate the intrinsic role of ZIP13 in adipocyte browning, we used C3H10T1/2 cells, which differentiate into brown/beige adipogenic lineages when exposed to a brown adipogenic cocktail. Depleting ZIP13 by RNAi significantly increased the expression of brown adipocyte markers (Fig 4A and 4B), which was further confirmed using another *Zip13* siRNA (S9A–S9D Fig). C/EBP-β protein levels were also increased by *Zip13* knockdown in C3H10T1/2 cells at the indicated time points (Fig 4C). The C/EBP-β protein level was investigated by a cycloheximide (CHX) chase experiment (Fig 4D and 4E), which suggested the possible involvement of ZIP13 in C/EBP-β protein stability.

We next investigated whether C/EBP-β ubiquitination was increased in *Zip13*-KO cells. Ubiquitinated C/EBP-β was detected by immunoprecipitation with the HA antibody, followed by immunoblotting with an anti-ubiquitin antibody. The level of ubiquitinated C/EBP-β protein was reduced by approximately half (0.48-fold decrease) in *Zip13*-KO cells compared with those in WT cells (Fig 4F), suggesting that C/EBP-β is resistant to ubiquitination in *Zip13*-KO cells, which might account for the increased adipocyte browning in these cells. To examine the biological relevance of C/EBP-β upregulation in *Zip13*-KO cells, we used retroviruses expressing short hairpin (sh) RNAs targeting C/EBP-β (shβ-1 and shβ-2) to downregulate C/EBP-β (Fig 4G), which efficiently blocked brown adipocyte differentiation (Fig 4H). These results indicated that the enhanced adipocyte browning due to loss of ZIP13 is caused by C/EBP-β accumulation.

### ZIP13-mediated zinc transport negatively regulates adipocyte browning

**Fig 5:**
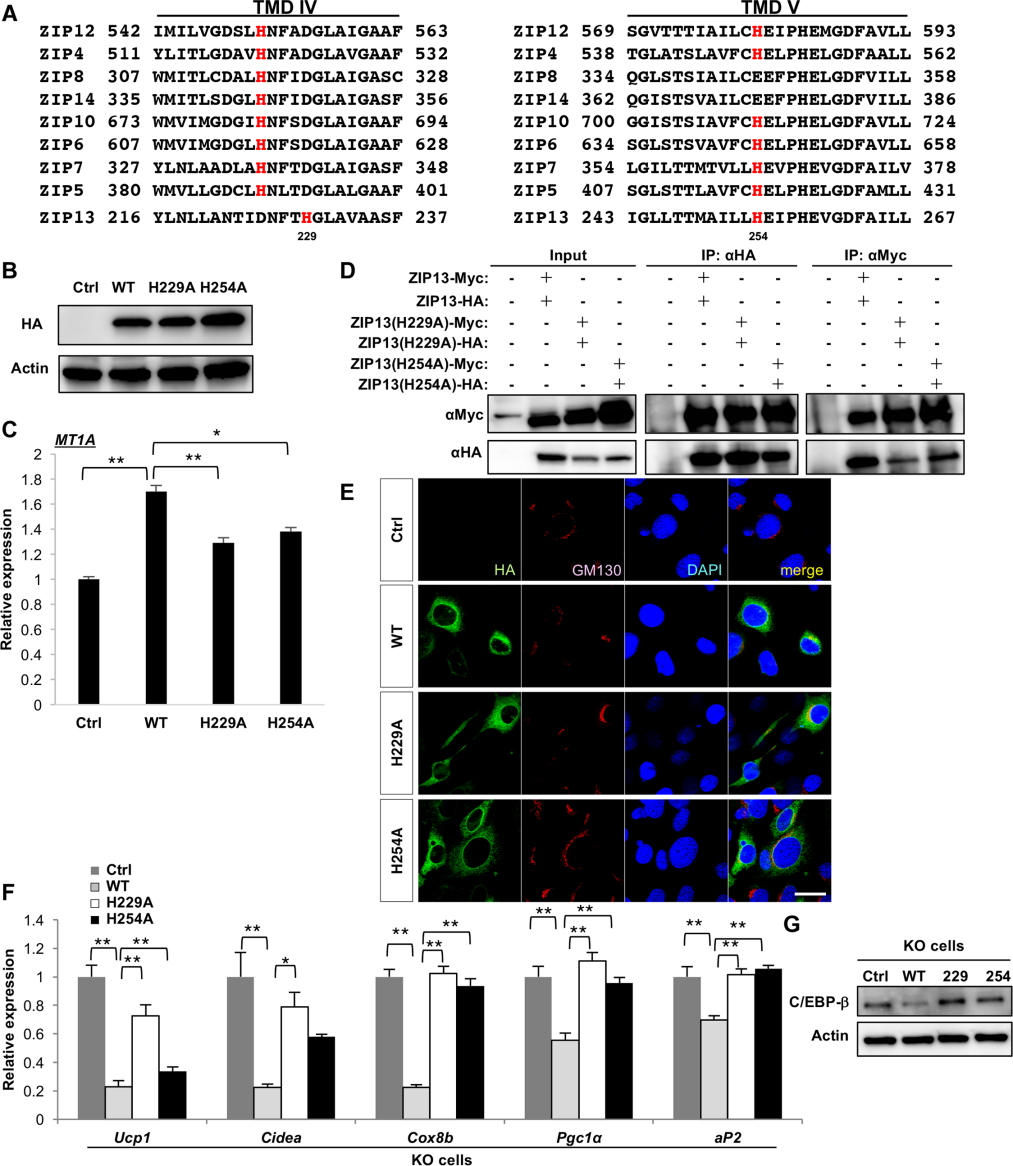
ZIP13-mediated zinc flux negatively regulates adipocyte browning. (A) Amino acid alignment of TMD IV and V among selected members of the mouse ZIP family. The His residues in TMD IV and V (red) are putative zinc-binding sites that are highly conserved among ZIP-family members. (B) Expression of WT ZIP13 and ZIP13 mutants (H229A and H254A) in C3H10T1/2 cells; β-actin is shown as a loading control. (C) *MT1A* gene expression in C3H10T1/2 cells expressing WT and mutant (H229A and H254A) ZIP13 (n = 4). We have showed the results that appeared to be statistically significant against the WT background. (D) Immunoprecipitation of HA- or Myc-tagged WT, H229A, or H254A ZIP13, followed by immunoblotting for HA- or Myc-tagged ZIP13 to detect the homophilic characteristics of the ZIP13 mutants H229A and H254A. (E) Subcellular localization of ZIP13-HA (WT, H229A, or H254A) expressed in *Zip13*-KO preadipocytes. Cells expressing HA-tagged WT, H229A, or H254A ZIP13 (left panels) were double-stained with the Golgi apparatus marker GM130 (middle panels); the merged images are shown on the right. Scale bars = 40 μm. (F) Expression levels of the indicated genes in *Zip13*-KO cells expressing Ctrl, WT ZIP13, or the H229A or H254A ZIP13 mutant (n = 4). (G) Expression of C/EBP-β protein 4 days after differentiation; β-actin is shown as a loading control. Error bars show SEM. *p<0.05, **p < 0.01.

We next investigated whether the zinc-transporting activity of ZIP13 is necessary for moderate adipocyte browning. The most highly conserved portions among the ZIP-family proteins are reported to be in transmembrane domains (TMDs) IV and V, which both contain common amino acids required for zinc binding, such as His (Fig 5A) [30]. To address whether these residues of ZIP13 contribute to intracellular zinc homeostasis, we generated a series of ZIP13 mutants (H229A and H254A) in which the His residues were replaced with Ala in TMDs IV and V (Fig 5B). As shown in Fig 5C, cells with exogenously expression of ZIP13 (WT) showed significantly upregulated *MT1A* mRNA levels, which correlates with cytosolic zinc levels, compared to the control (Ctrl); this result is consistent with previous reports [31,32] (Fig 5C). In contrast, exogenous expression of H229A or H254A mutant ZIP13 decreased the mRNA level of *MT1A* compared with ZIP13 (WT) (Fig 5C). Homophilic interactions (Fig 5D) and intracellular localization of these mutants were similar to those in the WT (Fig 5E), as previously reported [31]. Together, these findings indicated that H229 and H254 are important for increasing cytosolic zinc levels.

We next ectopically expressed the loss-of-function ZIP13 mutants in *Zip13*-KO cells in rescue experiments to determine whether they suppress the adipocyte browning induced by ZIP13 deficiency. Interestingly, expression of these loss-of-function ZIP13 mutants (H229A or H254A) could not suppress the adipocyte-browning phenotype (Fig 5F) or decrease C/EBP-β protein levels in *Zip13*-KO cells (Fig 5G). Finally, we examined whether zinc ion treatment could rescue the adipocyte-browning phenotype of *Zip13*-KO cells. Exogenous zinc ion treatment increased *MT1A* levels (S10A and S10B Fig) but did not reverse the adipocyte browning induced by ZIP13 deficiency (S10C Fig), indicating that specific zinc transport mediated by ZIP13 is indispensable for the proper homeostasis of adipocyte browning.

## Discussion

### ZIP13 is necessary for proper beige adipocyte differentiation in a cell-autonomous manner

The iWAT in *Zip13*-KO mice showed a browning phenotype that reflected increased energy expenditure (Fig 1A–1C and 1F, S1E Fig). Gene expression profiling showed that common fat genes were slightly upregulated in the iWAT of *Zip13*-KO mice, the expression of brown fat-specific genes were more notably increased, and the expression of white fat-specific genes was minimally affected (Fig 1E). These unbiased analyses demonstrated that ZIP13 is unlikely to be involved in the fate decision of white versus beige fat, but is likely involved in the inhibition of beige fat differentiation.

The differentiation of mesenchymal stem cells into adipocytes is regulated by a series of transcription factors that determine the sequence of events, such as commitment, differentiation, and activation [1]. For example, BMP7 and EBF2 are crucial for mesenchymal progenitor cells to commit to a brown or beige fat lineage [33,34]. Therefore, if these factors are activated in *Zip13*-KO cells, the expression of adipogenic regulators (such as *C/EBP-β*, *C/EBP-δ*, and *Krox20*) at the early stages should be upregulated. However, there was no change in the expression of these genes between WT and *Zip13*-KO cells (Fig 2E, S6B Fig). Instead, C/EBP-β protein levels were increased in *Zip13*-KO cells (Fig 2F), causing accelerated beige adipocyte differentiation. Exposure to cold, norepinephrine, or forskolin activates brown/beige adipocytes to express high levels of thermogenic genes [1]. In fact, we found that *Zip13*-KO cells exhibited increased thermogenic gene expression upon exposure to forskolin (Fig 2B), indicating that functional beige adipocytes are increased. However, the rate of increase in thermogenic gene expression was similar between *Zip13*-KO and WT cells (Fig 2B). These results suggested that ZIP13 is mainly involved in the differentiation rather than the activation of beige adipocytes.

We demonstrated that preadipocytes from *Zip13*-KO mice accelerate adipocyte browning at a higher rate than those from WT mice (Fig 2A–2C), and *Zip13* knockdown experiments showed similar results (Fig 4A and 4B). Furthermore, the exogenous expression of ZIP13 in *Zip13*-KO cells efficiently repressed adipocyte browning (Fig 2D). These results suggest that ZIP13 negatively regulates adipocyte browning in a cell-autonomous manner. We also showed that functional beige fat cells were increased in inguinal fat tissue of *Zip13*-KO mice (Fig 1A and 1C, S1E Fig), which might contribute to an increase in whole body VO_2_ of *Zip13*-KO mice (Fig 1F). Furthermore, the observation that the β3-adrenoreceptor agonist treatment increased whole-body energy expenditure even under thermoneutrality, indicated that beige fat of *Zip13*-KO mice at least partially contributed to the whole-body energy expenditure even if *Zip13*-KO mice were global KO mice. However, we cannot rule out the possibility that tissues other than beige fat might contribute to the increased in whole-body VO_2_ of *Zip13*-KO mice since *Zip13*-KO mice do have dermal defects [35], and this could play an important role in the increase in beige fat activity, as reported by Cannon and Nedergaard [36]. Further investigation is necessary to test this possibility, by analyzing tissue-specific *Zip13*-KO mice.

### ZIP13 is required for C/EBP-β homeostasis

C/EBP-β is involved in both adipogenesis and brown/beige adipocyte differentiation [12]. During adipogenesis, C/EBP-β induces the expression of C/EBP-α and PPARγ, the two major transcriptional inducers of adipogenic gene expression. *C/EBP-β*-KO mice have severely impaired brown fat development and reduced *Ucp1* expression [37]. Overexpression of C/EBP-β induces *Ucp1* expression in 3T3-L1 white adipocytes [38]. Upstream activators of C/EBP-β induce brown/beige fat differentiation [26,39–41]. In fact, browning is accelerated not only in cultured white but also brown *Zip13*-KO adipocytes, and in brown fat tissue of *Zip13*-KO mice fed a HFD (Fig 2, S4D, S4F and S11 Figs). Furthermore, *Zip13* knockdown increased adipocyte browning in C3H10T1/2 cells, which are capable of differentiating into the beige or brown adipocyte lineage when exposed to a brown adipogenic cocktail (Fig 4A–4C). These results suggest that *Zip13* may contribute to the browning of both white and brown adipocytes via the accumulation of C/EBP-β.

These findings raised the fundamental question of whether C/EBP-β stabilization provides a plausible explanation for the *Zip13* deficiency phenotype. We noted that white preadipocytes overexpressing C/EBP-β phenocopied *Zip13* deficiency with regard to accelerated adipocyte browning (Fig 3E and 3J, S8C and S8F Fig). Furthermore, the enhanced browning in *Zip13*-KO adipocytes was almost completely eliminated by C/EBP-β knockdown (Fig 4H). These results, obtained from overexpression or knockdown experiments, indicated that C/EBP-β stabilization is a crucial step for the enhanced browning resulting from ZIP13 deficiency.

An interesting observation in the present study was that C/EBP-β played specific roles in promoting beige adipocyte differentiation (Fig 3B–3E). C/EBP-β and PRDM16 comprise a transcriptional unit crucial for brown/beige fat differentiation; therefore, C/EBP-β protein induction might stabilize or recruit PRDM16 [42] and accelerate adipocyte browning. Indeed, PRDM16 expression was significantly increased in *Zip13*-KO cells compared with WT cells 4–6 days after the induction of differentiation (Fig 2F), supporting the idea that C/EBP-β protein accumulation stabilizes PRDM16.

### ZIP13-mediated zinc transport regulates C/EBP-β protein levels

ZIP13 is shown to transport zinc from the Golgi apparatus to the cytoplasm in mammalian cells [23,43]. Thus, we investigated whether this zinc-transporting ability of ZIP13 is required for inhibiting adipocyte browning. The conserved His residues in the TMDs are required for zinc transport in other ZIP-family proteins [44], and we demonstrated that these His residues in ZIP13 were crucial for ZIP13-mediated zinc transport for the inhibition of adipocyte browning (Fig 5F–5G). One possible explanation is that zinc transport elicited by a specific zinc transporter contributes to the stability of a particular protein, hence enabling it to perform its physiological function.

C/EBP-β is regulated by several post-translational modifications that are crucial for proper activation of the adipogenic program, such as phosphorylation, ubiquitination, and sumoylation [45,46]. We showed that the amount of ubiquitinated C/EBP-β was decreased in *Zip13*-KO cells (Fig 4F), causing accelerated adipocyte browning. There are two possible mechanisms underlying this observation. The first is that the expression or activity of deubiquitinating enzymes is upregulated; the second is that the ubiquitin system is downregulated. In fact, zinc blocks the activity of these deubiquitinating enzymes, including cysteine protease [47], and zinc is required for the activity of ubiquitin system-related proteins, including the RING-finger family E3 ligase [48]. Therefore, the activity of these enzymes may be altered in *Zip13*-deficient cells. Further analysis of *Zip13-*deficient cells will clarify the specific roles of C/EBP-β in beige adipocyte differentiation.

We also demonstrated that ZIP13-mediated zinc transport, but not a sufficient zinc supply in the form of zinc ions, is required for stabilizing C/EBP-β (Fig 5G, S10 Fig), suggesting that ZIP13-mediated zinc transport plays a specific role in clearing C/EBP-β proteins to inhibit adipocyte browning. ZIP13-mediated zinc transport might specifically determine the molecular fate of C/EBP-β partners, such as deubiquitinating enzymes or the RING-finger family E3 ligase, thereby affecting C/EBP-β stability and the rate of adipocyte browning. These hypotheses should be further verified by identifying the binding partners of ZIP13 via proteomic studies.

**Fig 6:**
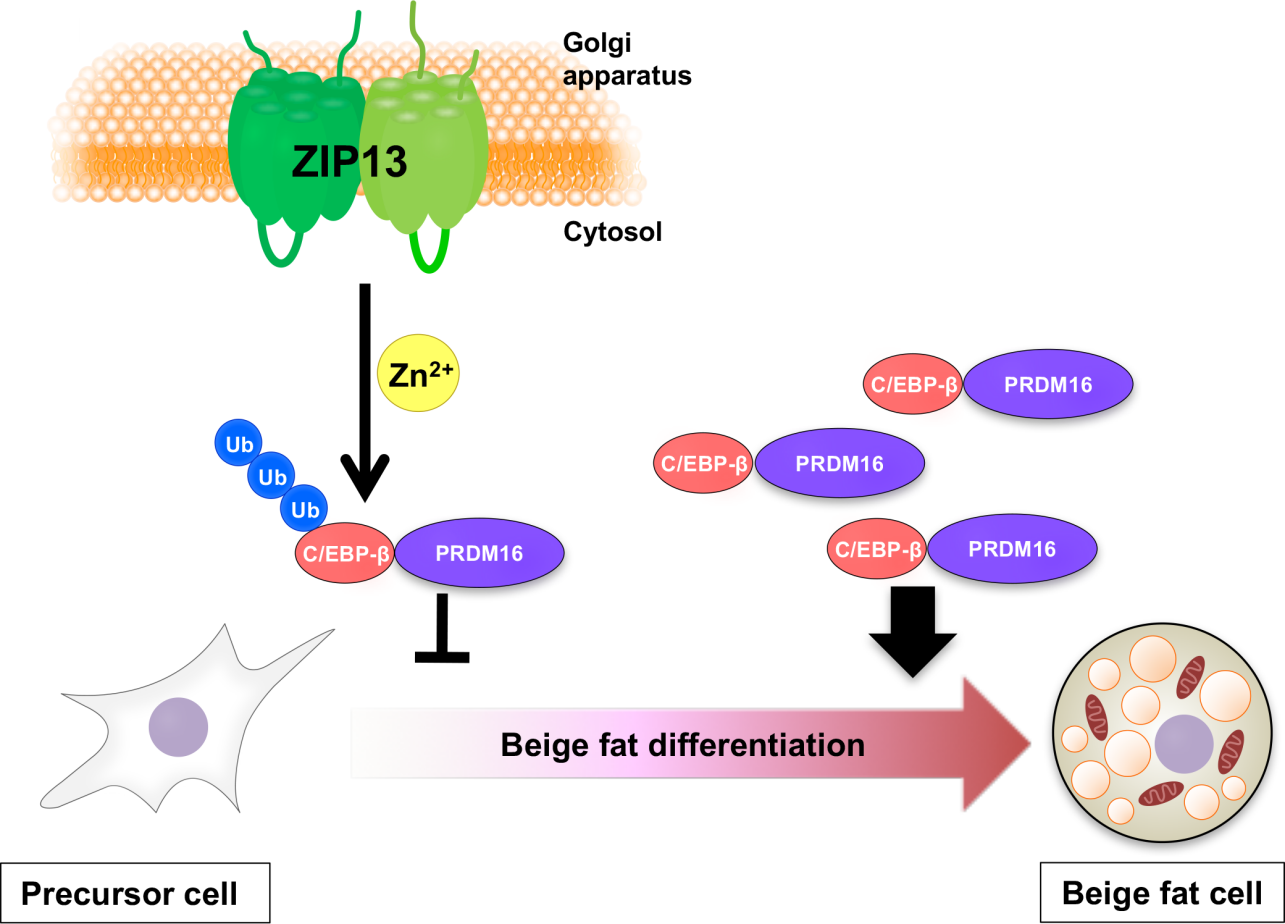
Schematic model of the role of ZIP13 in adipocyte browning. Zinc transport mediated by ZIP13 inhibits C/EBP-β accumulation, thereby negatively regulating adipocyte browning (left). Conversely, C/EBP-β accumulates in the *Zip13*-deficient condition (right).

In this study, while investigating the role of a causative gene for a human disease, we unexpectedly found a novel molecular adipocyte-browning mechanism regulated by the ZIP13-C/EBP-β signaling cascade (Fig 6). This system might be conserved in humans, considering the lean phenotype observed in a patient with a loss-of-function mutation of ZIP13 [23]. Elucidating the ZIP13-regulated adipocyte-browning pathway may contribute to the development of new therapeutics against obesity.

## Methods

### Animal studies

All mice were housed in specific pathogen-free barrier facilities, maintained under a 12-h light/dark cycle, given water *ad libitum*, and fed standard rodent feed (Oriental Yeast, Tokyo, Japan) or rodent feed containing 60% fat (Research Diet, New Brunswick, NJ, USA) from 8 to 14 weeks of age. As *Zip13-*KO mice show hypodontia [23], all mice used in the experiments were fed powdered feed to ensure adequate nutrition. All mice were backcrossed onto C57BL/6J mice for more than seven generations.

### Metabolic studies

The whole-body energy expenditure of *Zip13*-KO or WT mice at 10 weeks of age was measured using an ARCO 2000 mass spectrometer (Arco System, Chiba, Japan).

### Cell immortalization and culture

Immortalized white or brown preadipocytes were isolated from the iWAT or BAT of WT and *Zip13*-KO mice (6–8 weeks) by collagenase digestion, as described previously [49]. Preadipocytes were immortalized by retroviral transduction with the SV40T antigen and selection with puromycin (2 mg/mL). Immortalized preadipocytes were a mixed population and two cell lines were examined. Preadipocytes were seeded into collagen-coated dishes (Corning, Kennebunk, ME, USA) in DMEM/F12 (Gibco, Carlsbad, CA, USA) with 10% FCS. C3H10T1/2 cells were obtained from American Type Culture Collection. For white adipocyte differentiation, cells were induced with induction medium containing 10% FBS, 5 μg/mL insulin, 250 μM isobutylmethylxanthine (IBMX), and 2 μg/mL dexamethasone in DMEM. Two days after induction, the culture medium was changed to a maintenance medium containing 10% FBS and 5 μg/mL insulin. For the brown adipocyte cocktail, we used a formula described in previous reports [26–28]. Briefly, when the cells reached confluency, brown/beige adipocyte differentiation was induced by treating cells with DMEM containing 10% FBS, 250 μM IBMX, 2 μg/mL dexamethasone, 125 μM indomethacin, 5 μg/mL insulin, 1 nM T3, and 0.5 μM rosiglitazone. Two days after induction, the culture medium was changed to a maintenance medium containing 10% FBS, 5 μg/mL insulin, 1 nM T3, and 0.5 μM rosiglitazone. For cAMP treatment, cells were incubated with 10 μM forskolin for 4 h.

### RNAi-mediated gene knockdown

RNAi-mediated gene knockdown was performed as described previously [8]; siRNAs for *Zip13* were obtained from Invitrogen (Carlsbad, CA, USA) (silencer select siRNA [s206098] and stealth siRNA[MSS229105]).

### Immunoblotting and immunoprecipitation experiments

Immunoblotting and immunoprecipitation were performed as described previously [50]. The following antibodies were used for immunoblotting: anti-C/EBP-β (1:1,000; Cell Signaling, Danvers, MA, USA), anti-PRDM16 (1:1,000) [51], anti-PPARγ (1:1,000; Cell Signaling), anti-Rpb1CTD (RNA polymerase II CTD) (1:1000; Cell Signaling), anti-tubulin (1:3,000; Sigma-Aldrich, St Louis, MO, USA), or anti-β-actin (1:3,000; Sigma).

### Gene expression analysis

Total RNA was isolated from tissues using QIAzol (Qiagen, Valencia, CA, USA) following the manufacturer’s protocol. Reverse transcription reactions were performed using High Capacity cDNA Synthesis Kit (Applied Biosystems, Foster, CA, USA). The sequences of the primers used in this study are shown in S3 Table. Quantitative reverse transcriptase PCR (qRT-PCR) was performed with SYBR green fluorescent dye using an ABI 7500 Fast Real-Time PCR System. Relative mRNA expression was determined by relative standard curve methods using *18S* as an internal control to normalize samples.

### Microarray experiment

Samples were hybridized onto an array (Agilent SurePrint G3 Mouse GE 8x60K). The gene expression data set was deposited in the Gene Expression Omnibus database (GSE77933). Microarray analysis and functional enrichment analysis were as described in S1 Text.

### Plasmid construction and virus production

C-terminally HA-tagged plasmids expressing mouse ZIP13 (mZIP13-HA) were constructed by inserting cDNA into a pcDNA3.1, pMX-IRES-GFP, or pBabe-puro vector. Plasmids expressing zinc transport-incompetent ZIP13 mutants were constructed using two-step PCR. The plasmid expressing HA-C/EBP-β was kindly provided by Dr. Y. Kido (Kobe University) [52]. All constructs were verified by sequencing. Phoenix packaging (PLAT-E) cells, provided by Dr. T. Kitamura (Tokyo University), were transfected with retroviral vectors by lipofection [53]. After 48 h, the viral supernatant was collected and filtered. Cells were incubated for 6 h with the viral supernatant supplemented with 10 μg/mL polybrene.

### Immunohistochemistry and immunocytochemistry

Immunohistochemical analysis was performed as described previously [20,54,55], using an anti-Ucp1 antibody (1:250 dilution, Abcam, St. Charles, MO, USA). Immunocytochemistry was performed as described previously [50]. Anti-HA (1:100; MBL, Nagoya, Japan) and anti-GM130 antibodies (1:250; Transduction Lab, Lexington, KY, USA) were used for staining.

### Study approval

The protocol for animal experiments was approved by the Ethics Review Committee of Animal Experimentation of Juntendo University and Gunma University.

### Statistical analysis

All quantitative data were reported as the mean ± SEM. The Student’s *t*-test was performed for the comparison of two groups. For multiple comparisons, analysis of variance was performed by Two-way ANOVA followed by Bonferroni’s multiple comparison test or One-way ANOVA followed by Bonferroni’s multiple comparison test. A *p*-value of less than 0.05 was considered to indicate a statistically significant difference between two groups.

### Ethics statement

All mice were housed and cared for according to guidelines approved by the Animal Care and Use Committee of Juntendo University (280216), and by the Committee for Institutional Animal Care and Experimentation Committee at the Gunma University (16–048).

## Supporting information

S1 Fig Phenotype of adipose tissues in *Zip13*-KO mice. (A) Tissue weights of inguinal, epididymal, and brown fat tissues of WT and *Zip13*-KO mice. Tissue weights were normalized to whole-body weights. (B) The relative *Zip13* expression in various tissues from WT and *Zip13*-KO mice (n = 5–7). (C) H & E staining of epididymal fat in WT and *Zip13*-KO mice. Scale bars = 100 μm. (D) Expression of the indicated genes in epididymal fat tissue (n = 5–7). (E) Oxygen consumption rate (OCR) of inguinal fat tissue and brown fat tissue of WT and *Zip13*-KO mice at 24–26 weeks of age (n = 3). Data are shown as the mean ± SEM. *p < 0.05, **p < 0.01.
(TIF)

S2 Fig Characterization of upregulated or downregulated genes in *Zip13*-KO cells. (A) Genes that are upregulated or downregulated in *Zip13*-KO cells compared with WT cells. Color bar represents the gradient of log_2_-fold-changes in each comparison. (B) Gene Ontology Biological Processes (GOBPs) represented by the genes upregulated or downregulated by *Zip13* deletion. GOBPs are represented by enrichment scores, -log_10_(*p*), where *p* is the *p* value of the GOBPs that are enriched. (C) KEGG pathway enrichment represented by the genes upregulated or downregulated by *Zip13* deletion. The bars represent the enrichment scores, -log_10_(*p*), where *p* is the *p* value.
(TIF)

S3 Fig Metabolic phenotypes of *Zip13*-KO mice. (A) Average daily food intake of WT and *Zip13*-KO mice (n = 8–10). (B) Locomotor activity of WT and *Zip13*-KO at 18 weeks of age (n = 4–8). (C) Oxygen consumption rate of WT and *Zip13*-KO 10-week-old mice with CL316,243 (0.5mg/kg) under thermoneutral conditions (n = 5). Error bars are SEM. *p < 0.05, n.s., not significant.
(TIF)

S4 Fig Phenotypes of *Zip13*-KO mice fed a HFD. **(related to Fig 1)** (A) Tissue weights of inguinal, epididymal, and brown fat tissues of WT and *Zip13*-KO mice after 6 weeks on a HFD. Tissue weights were normalized to whole-body weights. (B) Insulin tolerance testing (left and middle) and the cumulative area under the curve between 0 min and 60 min for ITT (right) of 16-week-old WT and *Zip13*-KO mice fed an HFD for 8 weeks (n = 6–9). (C) Fasting blood glucose (left) and fasting plasm insulin (right) levels of 15-week-old WT and *Zip13*-KO mice fed an HFD for 7 weeks (n = 7–9). (D) H & E staining of inguinal fat tissue and brown fat tissue in WT and *Zip13*-KO mice fed a HFD. Scale bars = 200 μm. (E) Expression of the indicated genes in inguinal fat tissue (n = 6–7). (F) Expression of the indicated genes in brown fat tissue (n = 6–7). Data are shown as the mean ± SEM. *p < 0.05, **p < 0.01.
(TIF)

S5 Fig Phenotypes of *Zip13*-KO mice fed a HFD. (A) Body weights of mice from 5 to 9 weeks of age when fed a HFD (n = 7–8). (B)Lean mass (left) and Fat mass (right), (C) Lean mass/ Body weight (left) and Fat mass/Body weight (right) of WT and *Zip13*-KO mice fed an HFD for 6–7 weeks (n = 7–8). (D) CT evaluation of adiposity of WT and *Zip13*-KO mice after 6–7 weeks on a HFD. (E) CT evaluation of visceral fat mass and subcutaneous fat mass of WT and *Zip13*-KO mice after 6–7 weeks on a HFD. Tissue mass was normalized to whole-body weights. (F) Blood glucose concentrations were measured during the IPGTT in WT and *Zip13*-KO mice after 5–6 weeks on a HFD. Data are shown as the mean ± SEM. *p < 0.05, **p < 0.01.
(TIF)

S6 Fig Expression of adipogenic regulators at the early stages. (A) Western blots demonstrating the retroviral expression of ZIP13-HA in WT and *Zip13*-KO white preadipocytes; β-actin is shown as a loading control. (B) Time course (0, 1, 4, 8, 24, and 48 h) of mRNA expression of the indicated genes in differentiated white preadipocytes derived from WT and *Zip13*-KO mice. Data are shown as the mean ± SEM. *p < 0.05, **p < 0.01.
(TIF)

S7 Fig Expression of C/EBP-β. Western blot showing the stable HA-C/EBP-β expression in WT white preadipocytes; β-actin is shown as a loading control.
(TIF)

S8 Fig C/EBP-β overexpression accelerates adipocyte browning independent of adipogenesis. **(related to Fig 3)** (A) Schematic representation of the time course used in the following studies (B and C). (B) Full panel of indicated genes (related to Fig 3B and 3D). (C) Full panel of indicated genes (related to Fig 3C and 3E). (D) Schematic representation of the time course used in the following studies (E and F). (E) Full panel of indicated genes (related to Fig 3G and 3I). (F) Full panel of indicated genes (related to Fig 3H and 3J). Data are shown as the mean ± SEM. *p < 0.05, **p < 0.01.
(TIF)

S9 Fig *Zip13* knockdown increased adipocyte browning. (A) Diagram showing the time course of the experiments in B-D using C3H10T1/2 cells transfected with control siRNA (si-Ctrl) or *Zip13* siRNA (si-*Zip13*-#2). (B) *Zip13* expression decreased in C3H10T1/2 cells transfected with *Zip13* siRNA (n = 3). (C) Oil Red O staining of C3H10T1/2 cells transfected with si-Ctrl or si-*Zip13*-#2 under proadipogenic conditions. (D) The expression levels of the indicated genes were analyzed by qRT-PCR (n = 4). Data are shown as the mean ± SEM. *p < 0.05, **p < 0.01.
(TIF)

S10 Fig Zinc ion could not rescue the browning phenotype of *Zip13*-KO cells. (A) Schematic showing the time course used in B and C. WT and *Zip13*-KO (KO) white preadipocytes were differentiated using a brown-adipogenic induction cocktail with or without 100 μM ZnSO_4_. (B) *MT1A* expression in WT and KO cells (n = 4). (C) Expression levels of the indicated genes were measured by qRT-PCR (n = 4). Data are shown as the mean ± SEM. *p < 0.05, **p < 0.01. Please note that the expression level of indicated genes of both KO cells and its zinc-treated cells were significantly increased compared to WT cells or its zinc-treated cells, respectively.
(TIF)

S11 Fig ZIP13 negatively regulates brown adipocyte differentiation. (A) Expression of the indicated genes was measured by qRT-PCR (n = 4). (B) HA-tagged ZIP13 was expressed in *Zip13*-KO brown preadipocytes; β-actin was used as a loading control. (C) The brown preadipocytes derived from *Zip13*-KO mice expressing control (Ctrl) or HA-tagged ZIP13 (ZIP13) were differentiated. The mRNA expression levels of the indicated genes were measured by qRT-PCR (n = 4). (D) C/EBP-β protein expression at the indicated time points (0, 2, 4 and 6 days). WT and *Zip13*-KO brown preadipocytes were differentiated using a brown adipogenic cocktail at the indicated time points; Tubulin was shown as a loading control. Data are mean ± SEM. *p < 0.05, **p < 0.01.
(TIF)

S1 Table Upregulated and downregulated genes in *Zip13*-KO mice. (XLSX)

S2 Table Gene Ontology Biological Process (GOBP) analysis and Kyoto Encyclopedia of Genes and Genomes (KEGG) pathway enrichment analysis. (XLSX)

S3 Table Primer sequences. (PDF)

S1 Text Supplemental methods. (DOCX)
