# Supplementary figures and images for: Zinc transporter ZIP13 suppresses beige adipocyte biogenesis and energy expenditure by regulating C/EBP-β expression
Source: PLoS Genet. 2017 Aug 30;13(8):e1006950. doi: 10.1371/journal.pgen.1006950 (PMC5576661; doi:10.1371/journal.pgen.1006950)

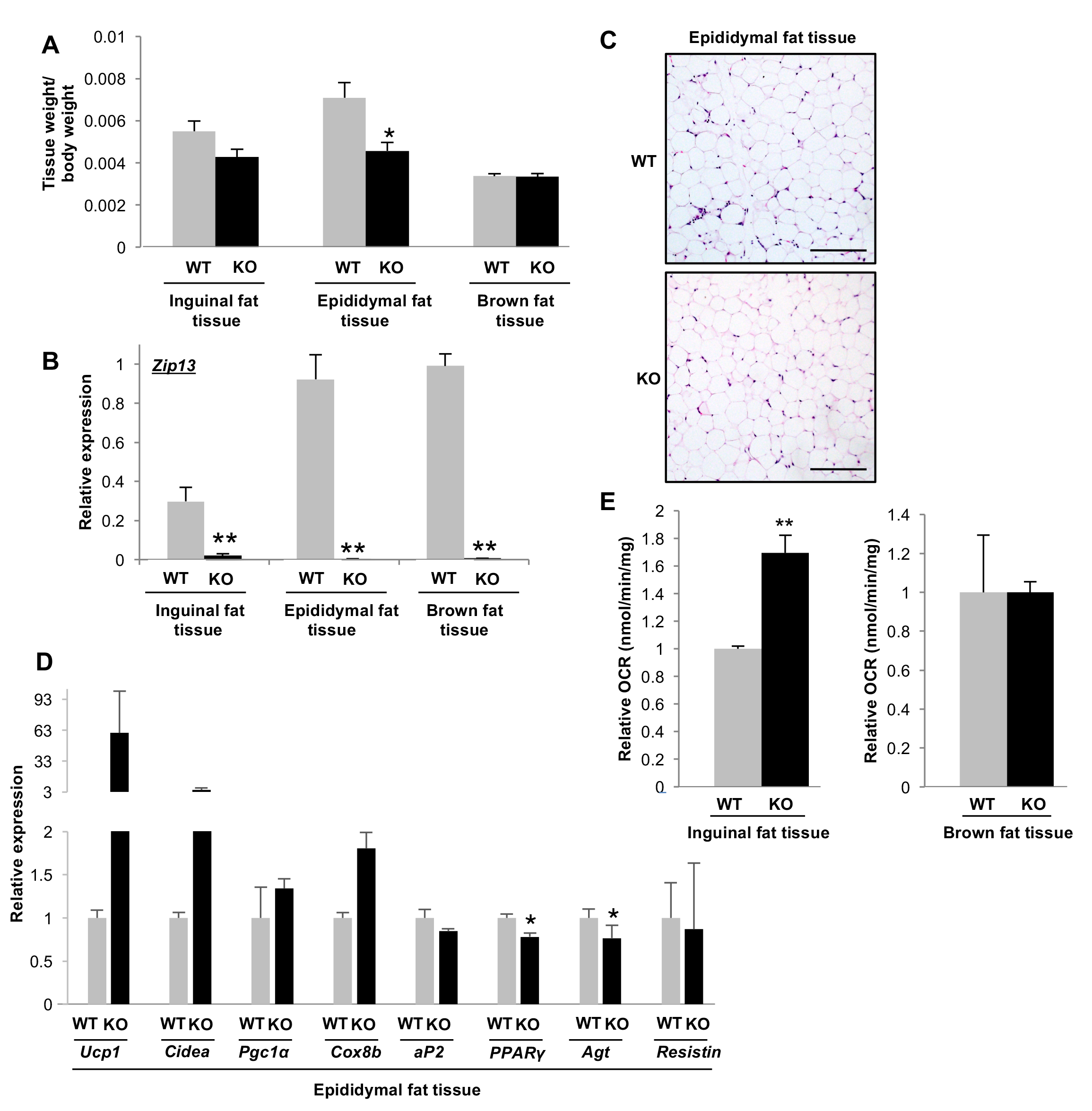

Supplement: S1 Fig — (A) Tissue weights of inguinal, epididymal, and brown fat tissues of WT and Zip13-KO mice. Tissue weights were normalized to whole-body weights. (B) The relative Zip13 expression in various tissues from WT and Zip13-KO mice (n = 5–7). (C) H & E staining of epididymal fat in WT and Zip13-KO mice. Scale bars = 100 μm. (D) Expression of the indicated genes in epididymal fat tissue (n = 5–7). (E) Oxygen consumption rate (OCR) of inguinal fat tissue and brown fat tissue of WT and Zip13-KO mice at 24–26 weeks of age (n = 3). Data are shown as the mean ± SEM. *p < 0.05, **p < 0.01. (TIF) [file pgen.1006950.s001.tif]

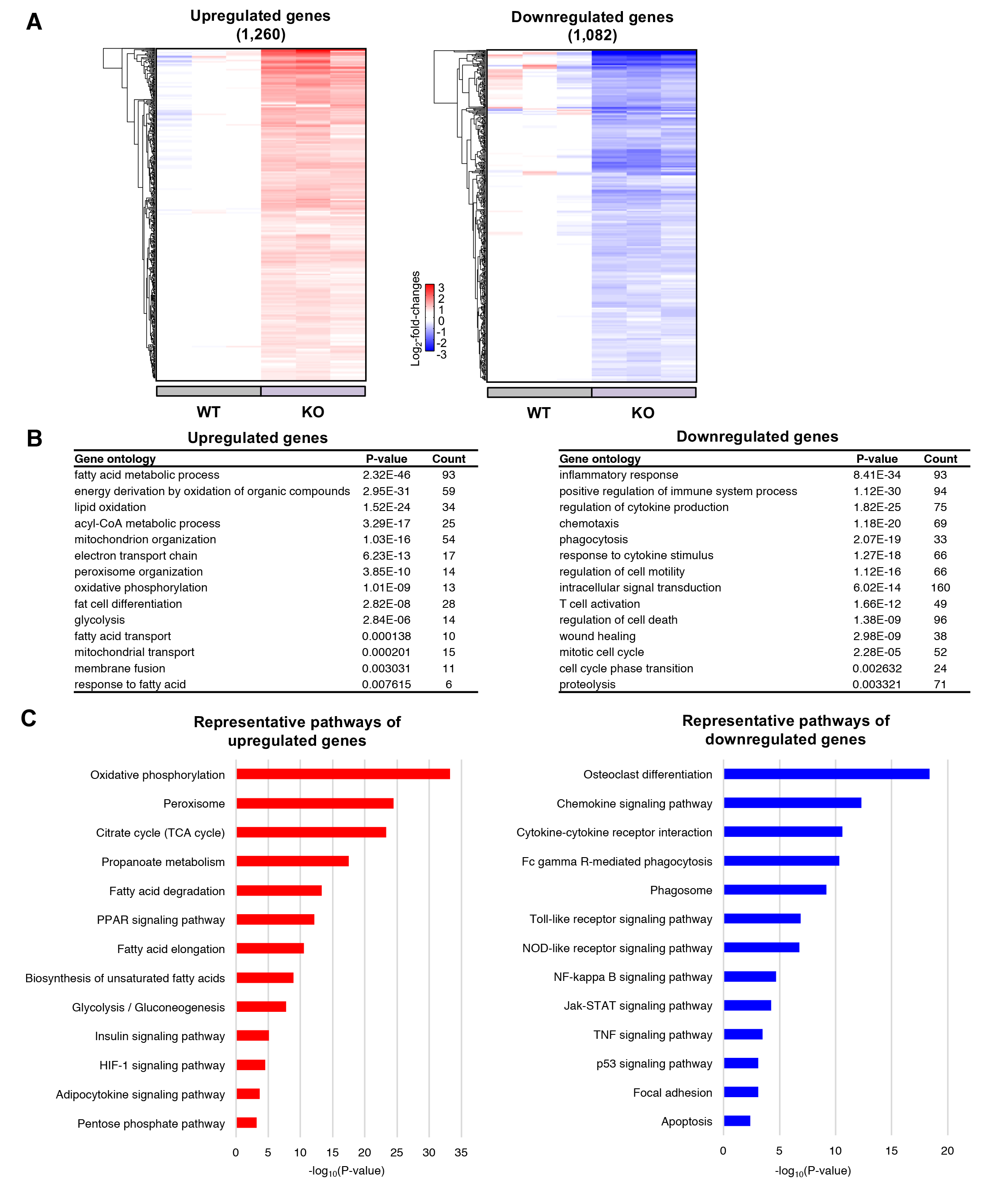

Supplement: S2 Fig — (A) Genes that are upregulated or downregulated in Zip13-KO cells compared with WT cells. Color bar represents the gradient of log2-fold-changes in each comparison. (B) Gene Ontology Biological Processes (GOBPs) represented by the genes upregulated or downregulated by Zip13 deletion. GOBPs are represented by enrichment scores, -log10(p), where p is the p value of the GOBPs that are enriched. (C) KEGG pathway enrichment represented by the genes upregulated or downregulated by Zip13 deletion. The bars represent the enrichment scores, -log10(p), where p is the p value. (TIF) [file pgen.1006950.s002.tif]

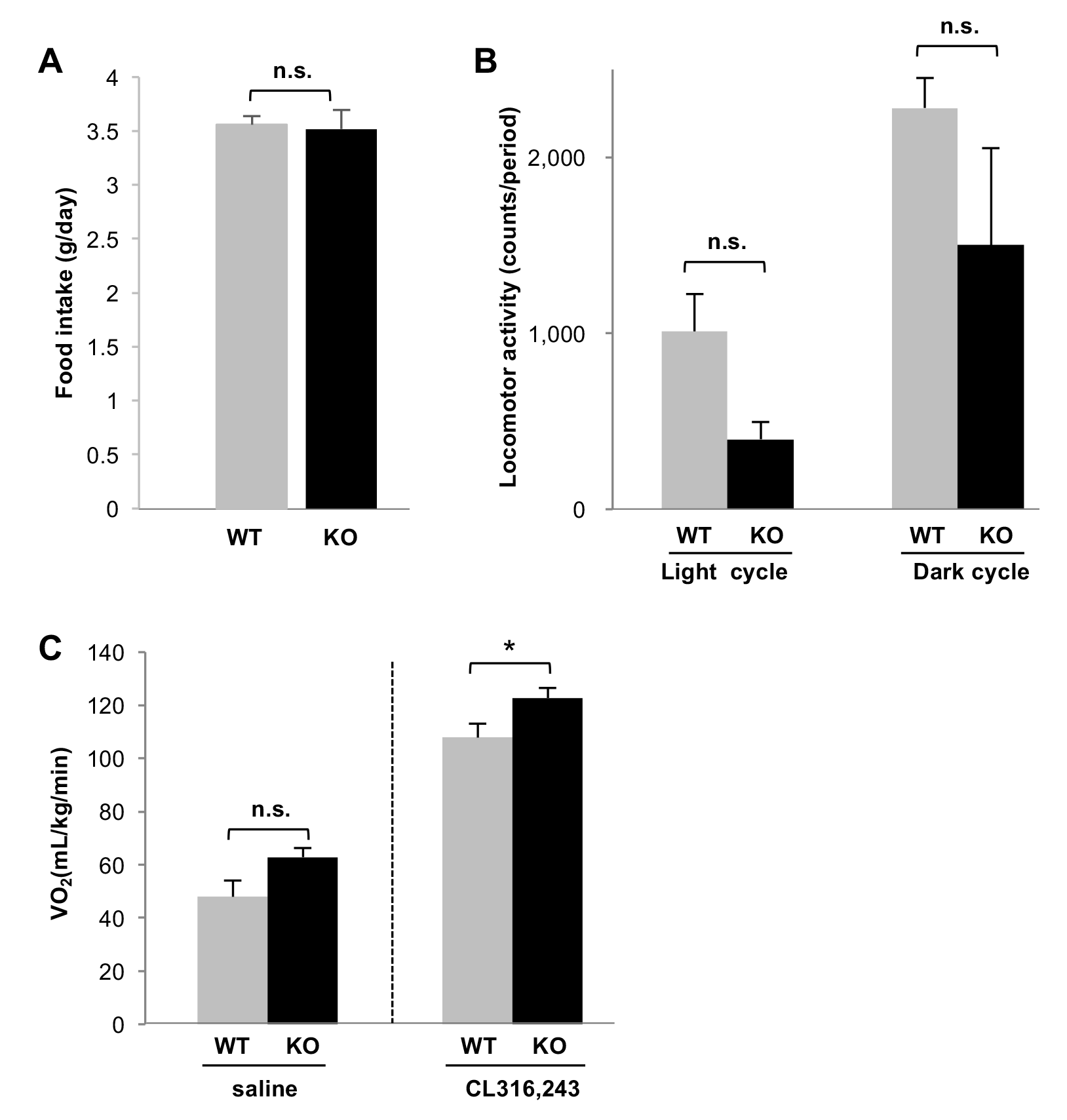

Supplement: S3 Fig — (A) Average daily food intake of WT and Zip13-KO mice (n = 8–10). (B) Locomotor activity of WT and Zip13-KO at 18 weeks of age (n = 4–8). (C) Oxygen consumption rate of WT and Zip13-KO 10-week-old mice with CL316,243 (0.5mg/kg) under thermoneutral conditions (n = 5). Error bars are SEM. *p < 0.05, n.s., not significant. (TIF) [file pgen.1006950.s003.tif]

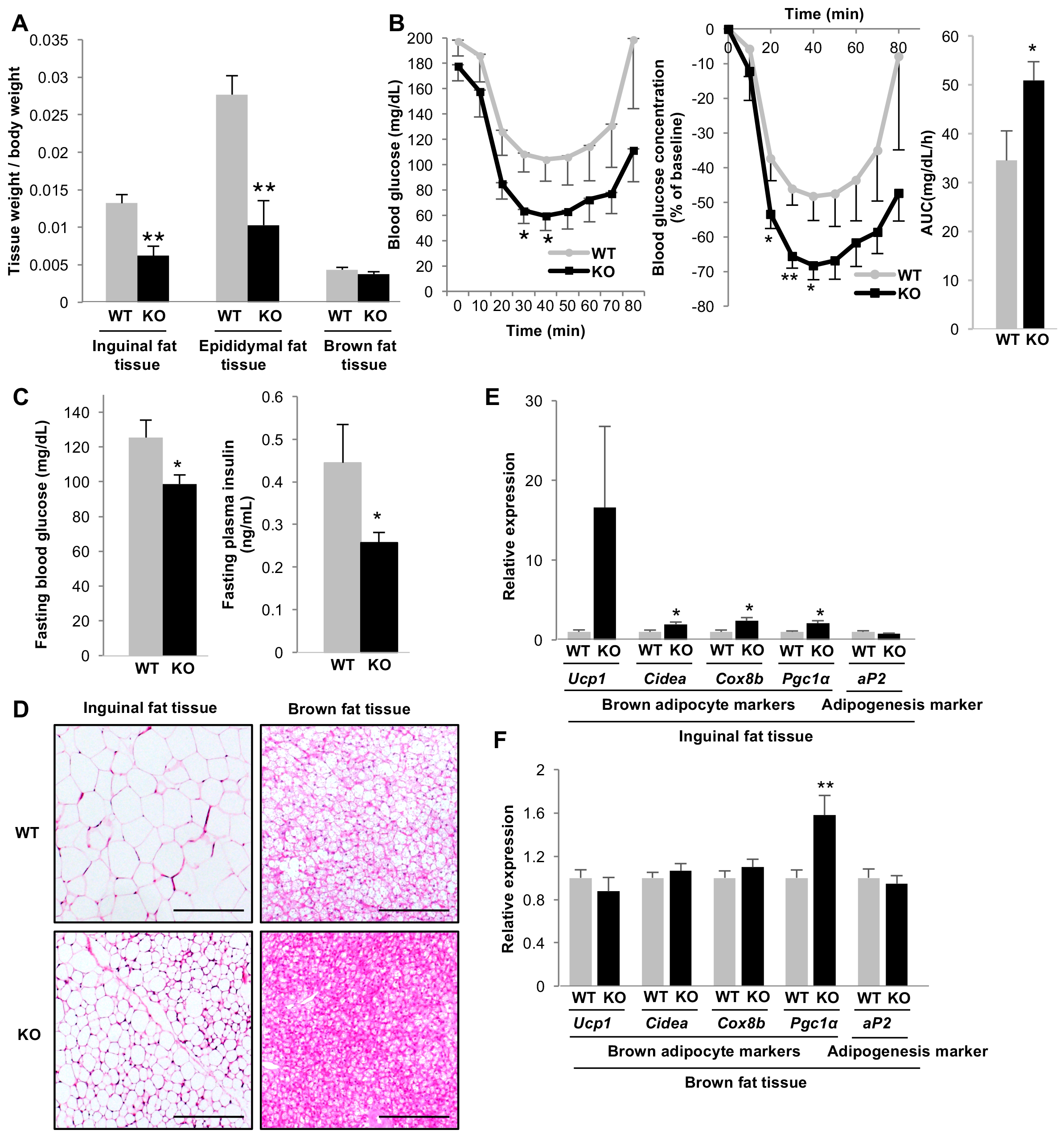

Supplement: S4 Fig — (related to Fig 1) (A) Tissue weights of inguinal, epididymal, and brown fat tissues of WT and Zip13-KO mice after 6 weeks on a HFD. Tissue weights were normalized to whole-body weights. (B) Insulin tolerance testing (left and middle) and the cumulative area under the curve between 0 min and 60 min for ITT (right) of 16-week-old WT and Zip13-KO mice fed an HFD for 8 weeks (n = 6–9). (C) Fasting blood glucose (left) and fasting plasm insulin (right) levels of 15-week-old WT and Zip13-KO mice fed an HFD for 7 weeks (n = 7–9). (D) H & E staining of inguinal fat tissue and brown fat tissue in WT and Zip13-KO mice fed a HFD. Scale bars = 200 μm. (E) Expression of the indicated genes in inguinal fat tissue (n = 6–7). (F) Expression of the indicated genes in brown fat tissue (n = 6–7). Data are shown as the mean ± SEM. *p < 0.05, **p < 0.01. (TIF) [file pgen.1006950.s004.tif]

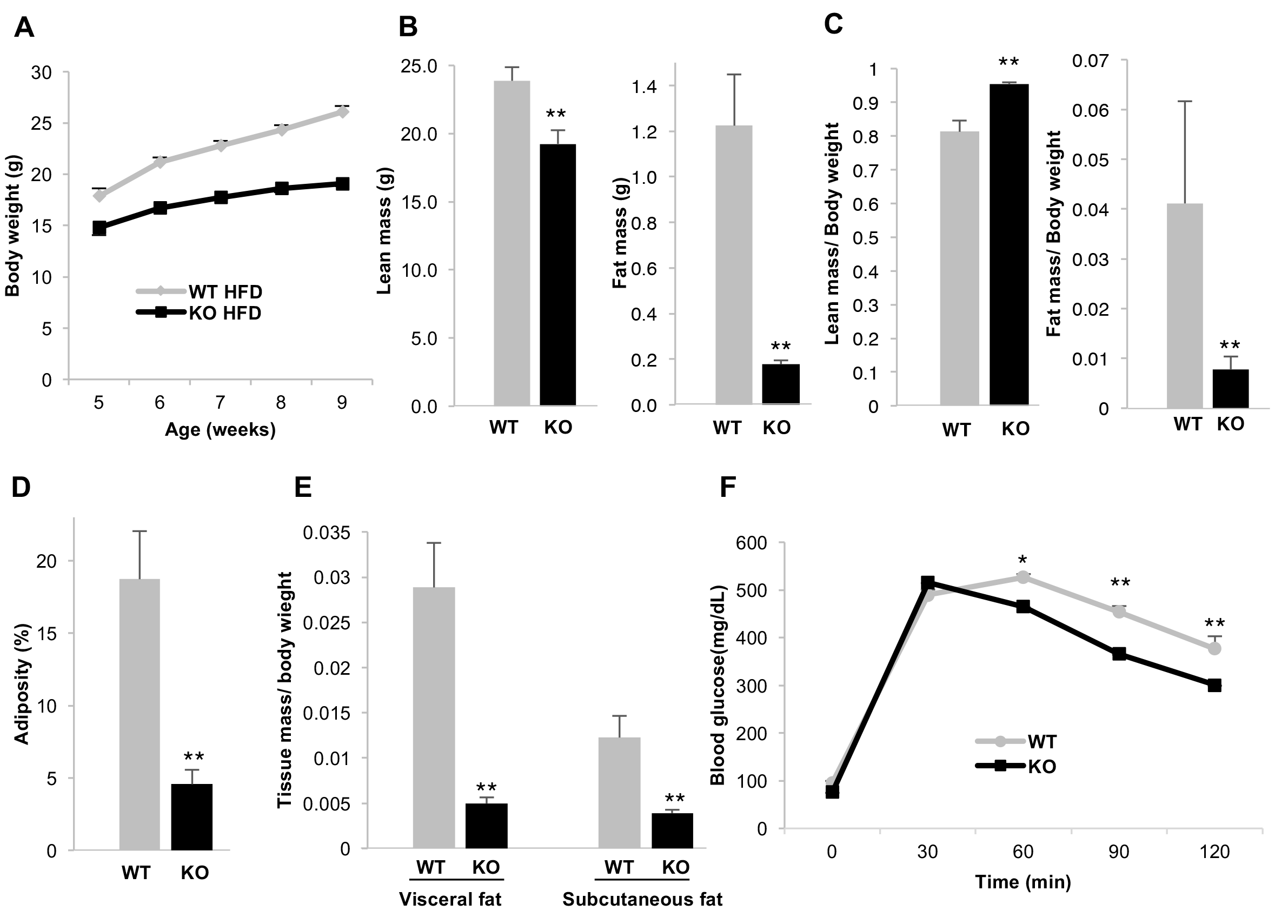

Supplement: S5 Fig — (A) Body weights of mice from 5 to 9 weeks of age when fed a HFD (n = 7–8). (B)Lean mass (left) and Fat mass (right), (C) Lean mass/ Body weight (left) and Fat mass/Body weight (right) of WT and Zip13-KO mice fed an HFD for 6–7 weeks (n = 7–8). (D) CT evaluation of adiposity of WT and Zip13-KO mice after 6–7 weeks on a HFD. (E) CT evaluation of visceral fat mass and subcutaneous fat mass of WT and Zip13-KO mice after 6–7 weeks on a HFD. Tissue mass was normalized to whole-body weights. (F) Blood glucose concentrations were measured during the IPGTT in WT and Zip13-KO mice after 5–6 weeks on a HFD. Data are shown as the mean ± SEM. *p < 0.05, **p < 0.01. (TIF) [file pgen.1006950.s005.tif]

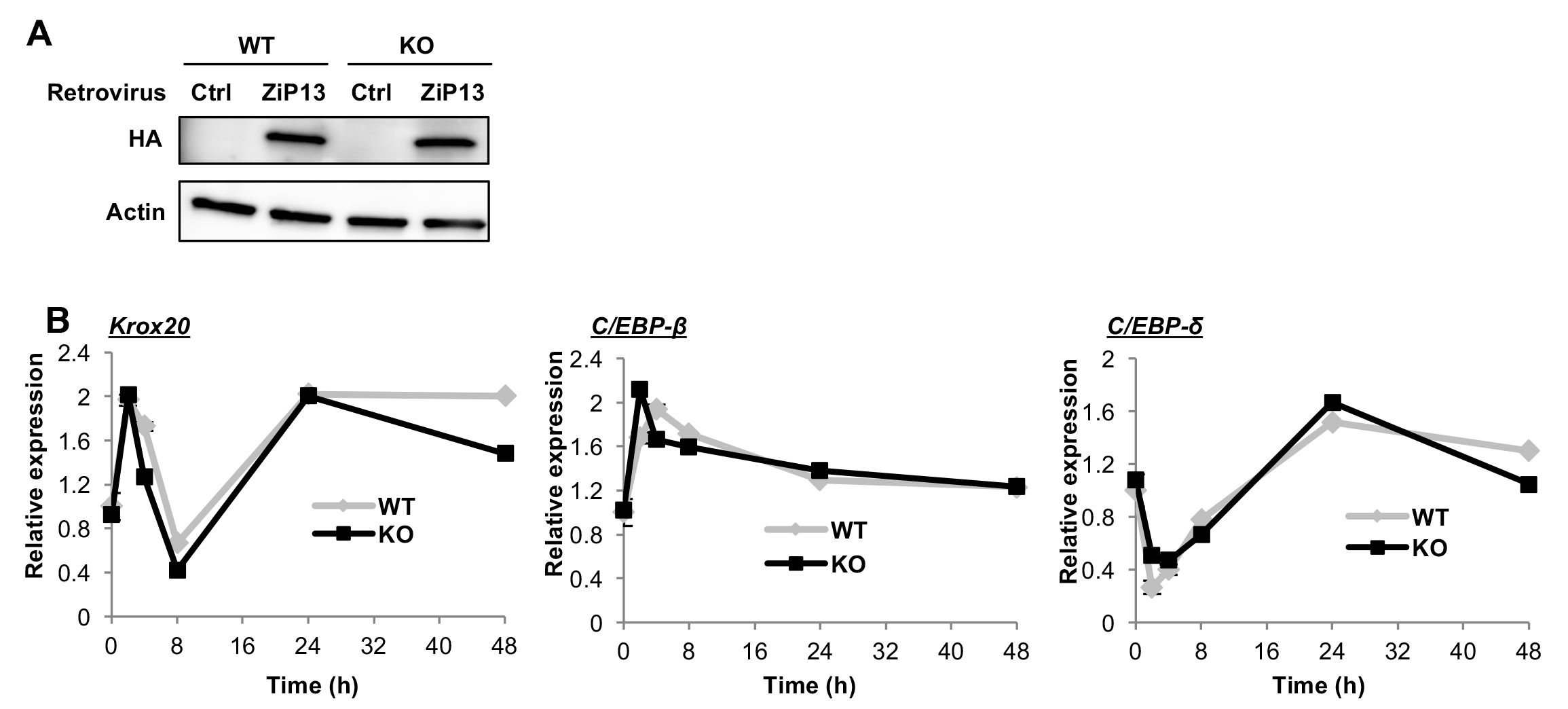

Supplement: S6 Fig — (A) Western blots demonstrating the retroviral expression of ZIP13-HA in WT and Zip13-KO white preadipocytes; β-actin is shown as a loading control. (B) Time course (0, 1, 4, 8, 24, and 48 h) of mRNA expression of the indicated genes in differentiated white preadipocytes derived from WT and Zip13-KO mice. Data are shown as the mean ± SEM. *p < 0.05, **p < 0.01. (TIF) [file pgen.1006950.s006.tif]

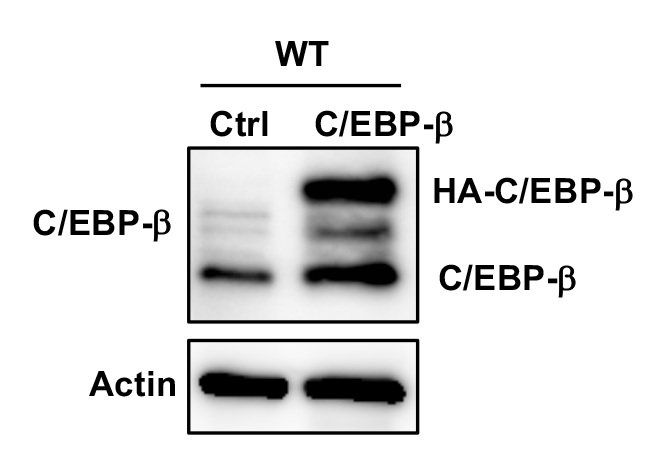

Supplement: S7 Fig — Western blot showing the stable HA-C/EBP-β expression in WT white preadipocytes; β-actin is shown as a loading control. (TIF) [file pgen.1006950.s007.tif]

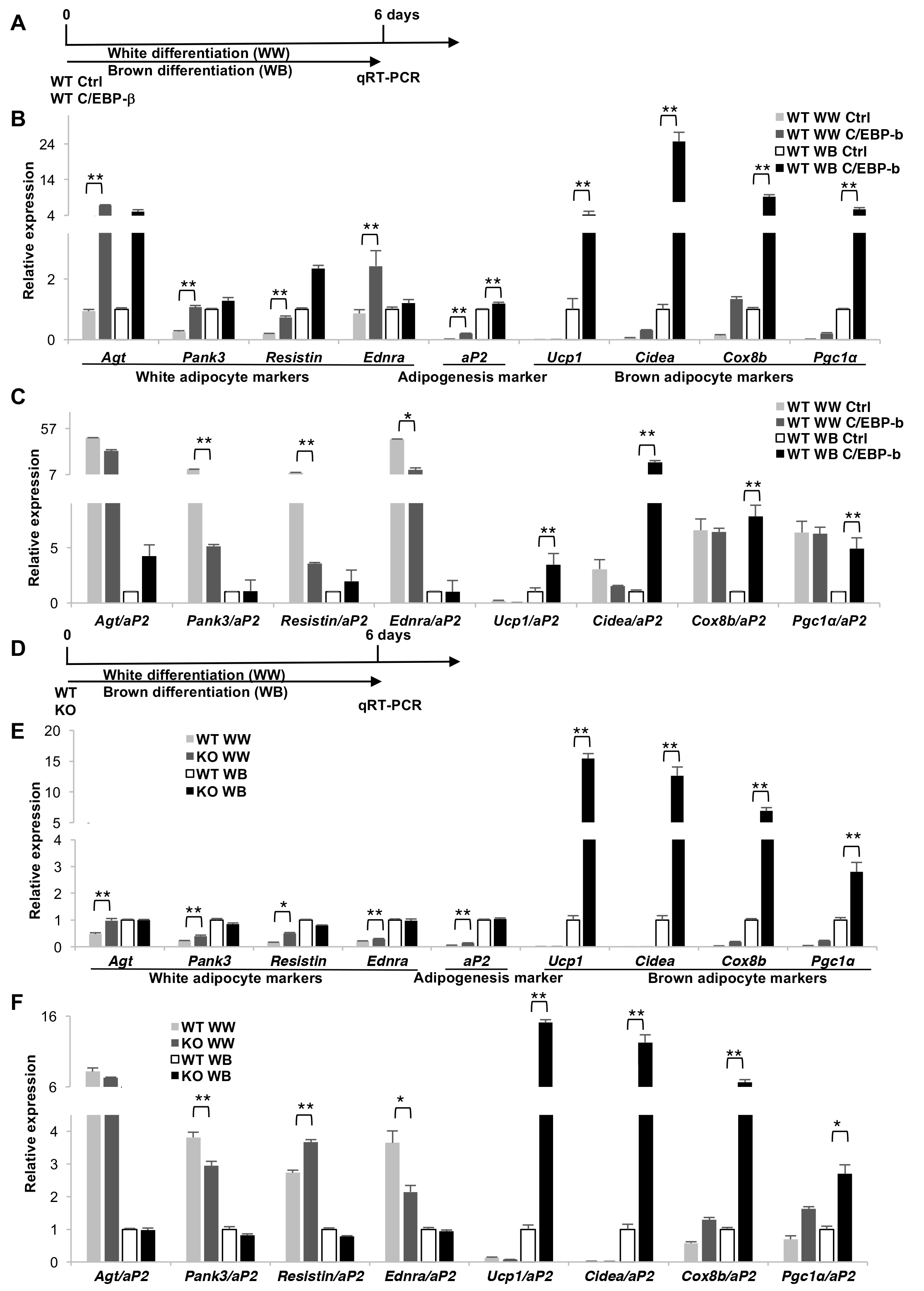

Supplement: S8 Fig — (related to Fig 3) (A) Schematic representation of the time course used in the following studies (B and C). (B) Full panel of indicated genes (related to Fig 3B and 3D). (C) Full panel of indicated genes (related to Fig 3C and 3E). (D) Schematic representation of the time course used in the following studies (E and F). (E) Full panel of indicated genes (related to Fig 3G and 3I). (F) Full panel of indicated genes (related to Fig 3H and 3J). Data are shown as the mean ± SEM. *p < 0.05, **p < 0.01. (TIF) [file pgen.1006950.s008.tif]

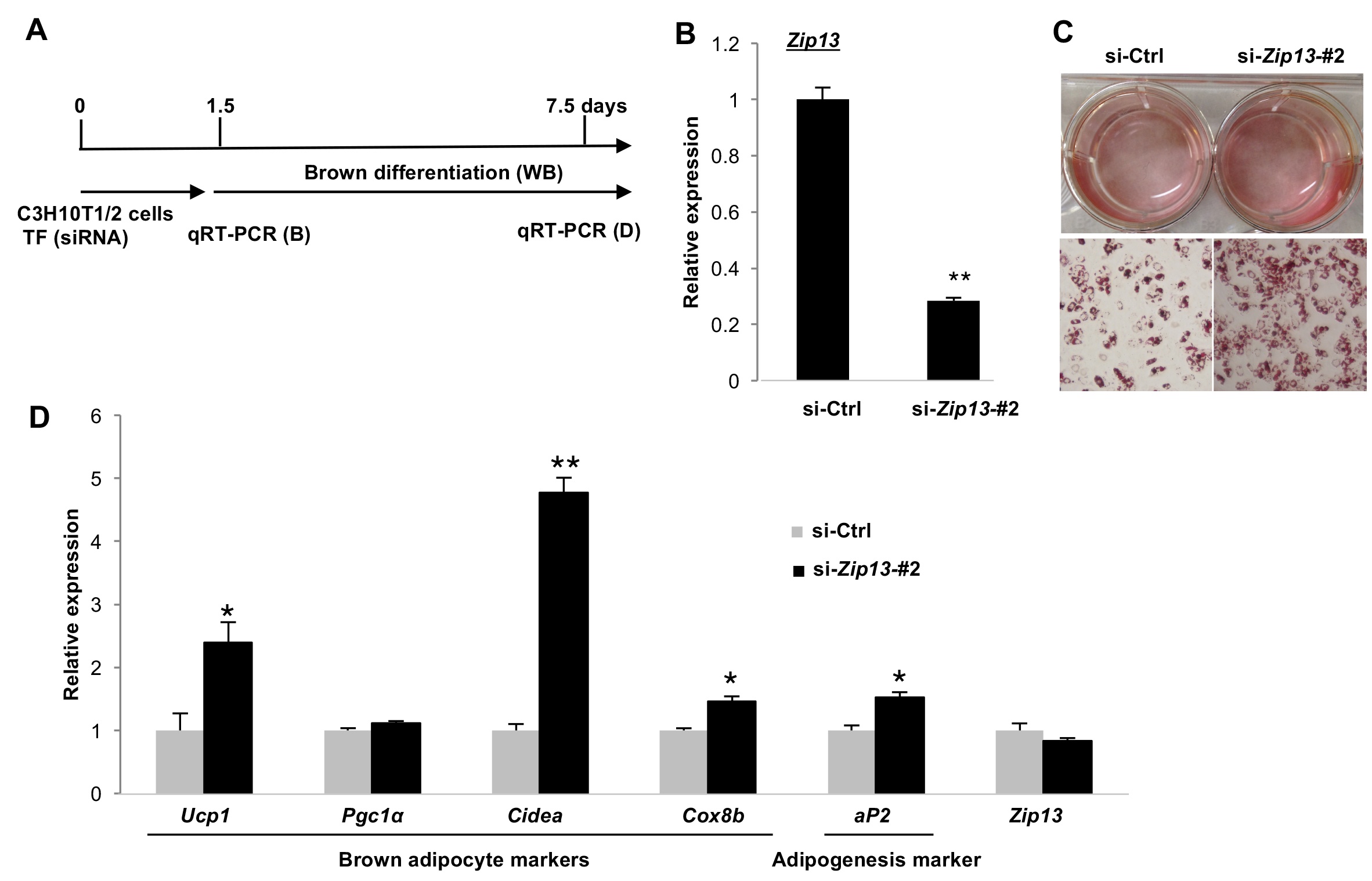

Supplement: S9 Fig — (A) Diagram showing the time course of the experiments in B-D using C3H10T1/2 cells transfected with control siRNA (si-Ctrl) or Zip13 siRNA (si-Zip13-#2). (B) Zip13 expression decreased in C3H10T1/2 cells transfected with Zip13 siRNA (n = 3). (C) Oil Red O staining of C3H10T1/2 cells transfected with si-Ctrl or si-Zip13-#2 under proadipogenic conditions. (D) The expression levels of the indicated genes were analyzed by qRT-PCR (n = 4). Data are shown as the mean ± SEM. *p < 0.05, **p < 0.01. (TIF) [file pgen.1006950.s009.tif]

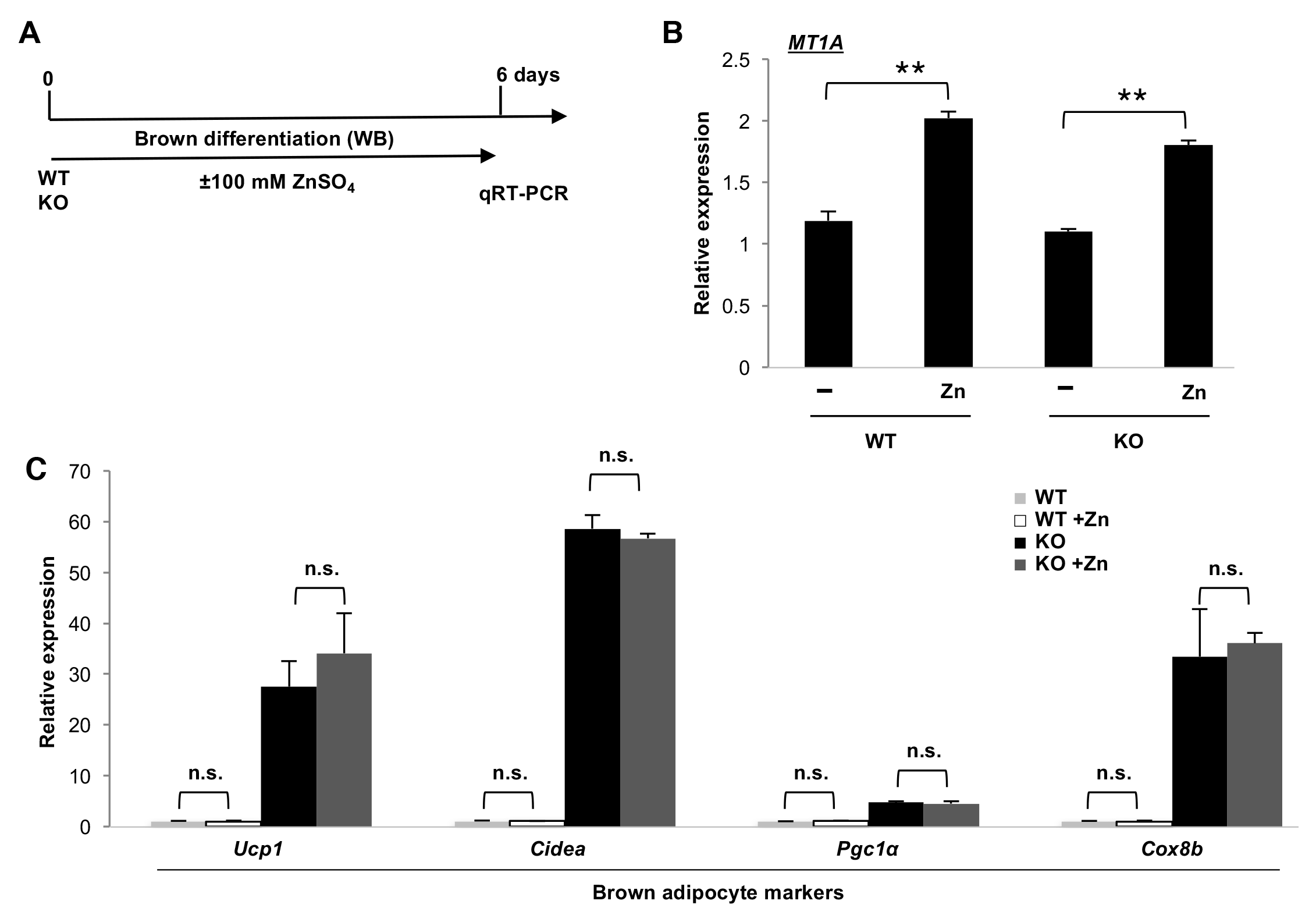

Supplement: S10 Fig — (A) Schematic showing the time course used in B and C. WT and Zip13-KO (KO) white preadipocytes were differentiated using a brown-adipogenic induction cocktail with or without 100 μM ZnSO4. (B) MT1A expression in WT and KO cells (n = 4). (C) Expression levels of the indicated genes were measured by qRT-PCR (n = 4). Data are shown as the mean ± SEM. *p < 0.05, **p < 0.01. Please note that the expression level of indicated genes of both KO cells and its zinc-treated cells were significantly increased compared to WT cells or its zinc-treated cells, respectively. (TIF) [file pgen.1006950.s010.tif]

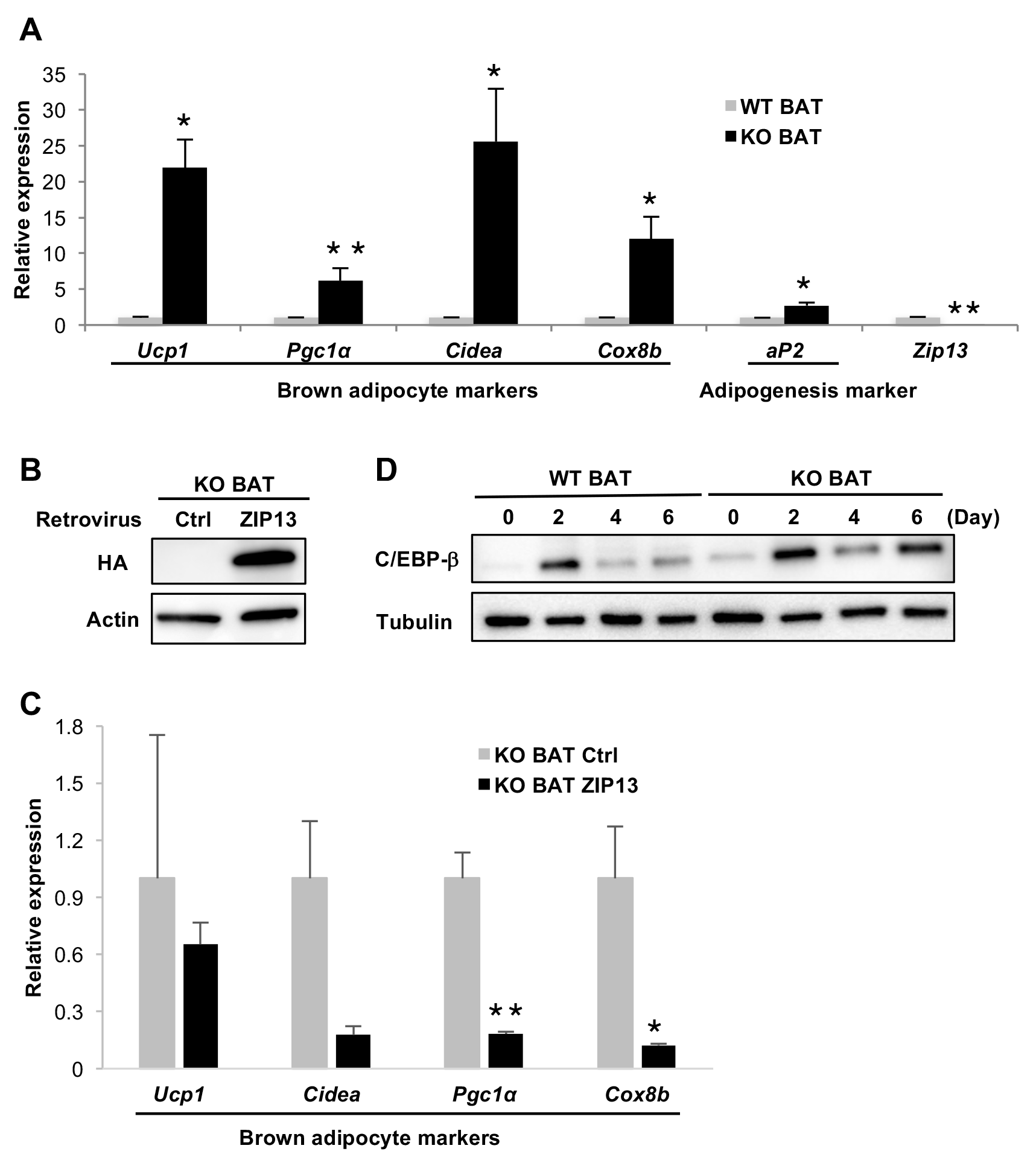

Supplement: S11 Fig — (A) Expression of the indicated genes was measured by qRT-PCR (n = 4). (B) HA-tagged ZIP13 was expressed in Zip13-KO brown preadipocytes; β-actin was used as a loading control. (C) The brown preadipocytes derived from Zip13-KO mice expressing control (Ctrl) or HA-tagged ZIP13 (ZIP13) were differentiated. The mRNA expression levels of the indicated genes were measured by qRT-PCR (n = 4). (D) C/EBP-β protein expression at the indicated time points (0, 2, 4 and 6 days). WT and Zip13-KO brown preadipocytes were differentiated using a brown adipogenic cocktail at the indicated time points; Tubulin was shown as a loading control. Data are mean ± SEM. *p < 0.05, **p < 0.01. (TIF) [file pgen.1006950.s011.tif]
