# Supplementary material for: Zinc transporter ZIP13 suppresses beige adipocyte biogenesis and energy expenditure by regulating C/EBP-β expression
Source: PLoS Genet. 2017 Aug 30;13(8):e1006950. doi: 10.1371/journal.pgen.1006950 (PMC5576661; doi:10.1371/journal.pgen.1006950)
Supplement: S3 Table — (PDF) [file pgen.1006950.s014.pdf]

**Supplemental table**

**Table S3. Primer sequences**

| Gene | Species | Forward primer | Reverse primer |
|---|---|---|---|
| *18S* | mouse | TTCTGGCCAACGGTCTAGACAAC | CCAGTGGTCTTGGTGTGCTGA |
| *aP2* | mouse | ACACCGAGATTTCCTTCAAACTG | CCATCTAGGGTTATGATGCTCTTCA |
| *PPAR$\gamma$* | mouse | GTGCCAGTTTCGATCCGTAGA | GGCCAGCATCGTGTAGATGA |
| *Cidea* | mouse | ATCACAACTGGCCTGGTTACG | TACTACCCGGTGTCCATTTCT |
| *Cox8b* | mouse | GAACCATGAAGCCAACGACT | GCGAAGTTCACAGTGGTTCC |
| *Pgc1$\alpha$* | mouse | AGCCGTGACCACTGACAACGAG | GCTGCATGGTTCTGAGTGCTAAG |
| *Prdm16* | mouse | TGGCCTTCATCACCTCTCTGAA | TTTCTGATCCACGGCTCCTGTGA |
| *Ucp1* | mouse | CACCTTCCCGCTGGACACT | CCCTAGGACACCTTTATACCTAATGG |
| *C/EBP-$\beta$* | mouse | AAGCTGAGCGACGAGTACAAGA | GTCAGCTCCAGCACCTTGTG |
| *C/EBP-$\delta$* | mouse | CGACTTCAGCGCCTACATTGA | CTAGCGACAGACCCCACAC |
| *Krox20* | mouse | TGACTATTGTGGCCGCAAGTT | TTCTGCCGAAGGTGGATCTT |
| *Resistin* | mouse | AAGAACCTTTCATTTCCCCTCCT | GTCCAGCAATTTAAGCCAATGTT |
| *Agt* | mouse | GCACCCTGGTCTCTTTCTACC | TGTGTCCATCTAGTCGGGAGG |
| *Pank3* | mouse | TGCTGTAGTGTCCCATTTCTGCCT | AGCTGGAACAGCAACACCTAGGAA |
| *Ednra* | mouse | AGCAGGAAGGTGTGCTGACT | GTAGGAGGCATCTGGGTTGA |
| *Zip13* | mouse | AGGCCCCCAGCAAAGACCCCA | CTTTTTGCTCACAAGGAAGCT |
| *MT1A* | mouse | GCCTGCAAGAACTGCAAGTG | ATAGGAAGACGCTGGGTTGG |
