# Supplementary material for: Zinc transporter ZIP13 suppresses beige adipocyte biogenesis and energy expenditure by regulating C/EBP-β expression
Source: PLoS Genet. 2017 Aug 30;13(8):e1006950. doi: 10.1371/journal.pgen.1006950 (PMC5576661; doi:10.1371/journal.pgen.1006950)
Supplement: S1 Text — (DOCX) [file pgen.1006950.s015.docx]

**Supplemental methods**

**Gene expression analysis.**

Total RNA was isolated from tissues using Qiazol (Qiagen, Valencia, CA) following the manufacturer’s protocol. Reverse transcription reactions were performed using the High Capacity cDNA Synthesis Kit (Applied Biosystems, Foster, CA). S3 Table shows the primer sequences used in this study. Quantitative reverse transcriptase PCR (qRT-PCR) was performed with SYBR green fluorescent dye using an ABI 7500 Fast Real-Time PCR System. Relative mRNA expression was determined by relative standard curve methods using *18S* as an internal control to normalize the samples.

**Microarray analysis**.

We first normalized the log_2_ intensities of all probes using the quantile normalization method [[1](#_ENREF_1)]. Before identifying differentially expressed genes (DEGs) (genes that were up- or downregulated in *Zip13*-KO compared with WT cells), we first confirmed the expression of individual probes using a Gaussian-mixture modeling method as previously described [[2](#_ENREF_2)]. We used normalized log_2_ intensities to identify DEGs when comparing WT and *Zip13*-KO cells using a previously reported integrative statistical hypothesis testing method that computes adjusted *p* values (*p*) by combining a two-tailed t-test and a median-ratio test [[3](#_ENREF_3)]. Genes with *p*< 0.05 and log_2_-median-ratios > 0.58 (1.5-fold) among the expressed genes were identified as DEGs.

**Functional enrichment analysis.**

To identify cellular processes or pathways represented by the DEGs, we performed functional enrichment analysis for the DEGs using ConsensusPathDB [[4](#_ENREF_4)] and selected the GPBPs (level 3 and 4) and KEGG pathways with a *p* value < 0.01 and gene count ≧ 5 as the ones represented by the DEGs.

**Body composition analysis and locomotor activity.**

Body fat percentage, visceral fat mass and subcutaneous fat mass were measured using an X-ray CT scanner (LaTheta LCT-200; Hitachi Aloka Medical, Tokyo, Japan). Locomotor activity was measured in a metabolic chamber using an infrared light beam detection system (ACTIMO-100; Shinfactory, Fukuoka, Japan). Total locomotor activity during a 12-h period was measured.

**Measurement of blood glucose levels and insulin levels.**

The intraperitoneal glucose tolerance test (IPGTT) and insulin tolerance test (ITT) were performed as described previously [5]. For IPGTT, animals were fasted for 13 h, and then injected intraperitoneally with 2 g/kg glucose. Glucose levels were measured using a glucose analyzer (Glutest mint, Sanwa Chemical Co., Japan). Insulin levels were measured by enzyme-linked immunosorbent assay (ELISA) kit (Morinaga Co., Kanagawa, Japan). For the ITT, mice were injected intraperitoneally with insulin (0.75 units/kg).

**Tissue respiration assay and cellular respiration assay.**

Tissue respiration was analyzed using a Clark-type oxygen electrode (YSI Incorporated, Yellow Springs, OH). Freshly isolated inguinal WAT and interscapular BAT tissues were minced until homogenous. Oxygen consumption rate (OCR) was normalized to protein concentration measured using the micro BCA protein assay kit (Pierce Protein Biology, Thermo Fisher Scientific, Waltham, MA). For the cellular respiration assay, immortalized white adipocytes were differentiated using a brown adipogenic cocktail. On day 6 of differentiation, adipocytes were detached and re-seeded at 2 x 10^4^ cells/well into XFe24 cell culture microplates (Seahorse Bioscience). OCR was measured using an XFe24 Extracellular Flux Analyzer. Oligomycin was used to determine uncoupled respiration.

**References**

1. Bolstad BM, Irizarry RA, Astrand M, Speed TP (2003) A comparison of normalization methods for high density oligonucleotide array data based on variance and bias. Bioinformatics 19: 185-193.

2. Lee HJ, Suk JE, Patrick C, Bae EJ, Cho JH, et al. (2010) Direct transfer of alpha-synuclein from neuron to astroglia causes inflammatory responses in synucleinopathies. J Biol Chem 285: 9262-9272.

3. Hwang D, Smith JJ, Leslie DM, Weston AD, Rust AG, et al. (2005) A data integration methodology for systems biology: experimental verification. Proc Natl Acad Sci U S A 102: 17302-17307.

4. Kamburov A, Wierling C, Lehrach H, Herwig R (2009) ConsensusPathDB--a database for integrating human functional interaction networks. Nucleic Acids Res 37: D623-628.

5. Shigihara N, Fukunaka A, Hara A, Komiya K, Honda A, et al. (2014) Human IAPP-induced

pancreatic beta cell toxicity and its regulation by autophagy. J Clin Invest 124: 3634-3644.
